# Nanoarchitectonics of Metal–Organic Framework on Fullerene Assemblies: Fabrication of Hierarchical Nanostructured Carbon Electrocatalysts

**DOI:** 10.1021/acsami.6c03512

**Published:** 2026-05-15

**Authors:** Rabindra Nath Acharyya, Biswa Nath Bhadra, Sabina Shahi, Kenji Hayashida, Shusaku Fujita, Kotaro Takeyasu, Katsuhiko Ariga, Lok Kumar Shrestha

**Affiliations:** † Research Center for Materials Nanoarchitectonics (MANA), 52747 National Institute for Materials Science (NIMS), 1-1 Namiki, Tsukuba, Ibaraki 305-0044, Japan; ‡ Graduate School of Science and Technology, 13121 University of Tsukuba, 1-1-1 Tennodai, Tsukuba, Ibaraki 305-8573, Japan; § Institute Charles Gerhardt Montpellier (ICGM), Centre National de la recherche scientifique (CNRS), Montpellier 34095, France; ∥ Graduate School of Environmental Science, 12810 Hokkaido University, N10W5, Sapporo 060-0810, Hokkaido, Japan; ⊥ Institute for Catalysis, Hokkaido University, N21W10, Sapporo 001-0021, Hokkaido, Japan; # Department of Materials Science, Institute of Pure and Applied Sciences, University of Tsukuba, 1-1-1 Tennodai, Tsukuba, Ibaraki 305-8573, Japan; ¶ Department of Advanced Materials Science, Graduate School of Frontier Sciences, The University of Tokyo, 5-1-5 Kashiwanoha, Kashiwa 277-8561, Chiba, Japan

**Keywords:** MOF-on-fullerene, layer-by-layer, hierarchical nanostructure, acidic ORR, durability

## Abstract

Designing efficient and durable carbon-based electrocatalysts remains a major challenge for next-generation energy technologies. Here, we introduce a comprehensive nanoarchitectonics approach for constructing metal–organic framework on fullerene (MOFOF) hybrids with diverse dimensionalities spanning 0D nanospheres, 1D nanorods and nanotubes, 2D sheets, and 3D cubic assemblies, and demonstrate their potential for electrocatalyst development through the pyrolysis of a selected hybrid. Through a sequential layer-by-layer (or, step-by-step) growth of a typical MOF (ZIF-67) on self-assembled fullerene supports, we generated a family of MOFOF nanostructures with diverse morphologies. Subsequent high-temperature carbonization and ammonia-assisted nitrogen doping are expected to transform these MOFOF precursors into hierarchical nitrogen-doped Co/C electrocatalysts featuring well-defined Co–N_*x*_ active sites embedded within graphitized carbon frameworks. Using quasi-1D fullerene nanotubes (FNTs) as a structural template produced the most efficient catalyst, Co–N@CT-900, which delivered an onset potential of 0.78 V vs RHE and a Tafel slope of 56.6 mV dec^−1^, along with excellent electrochemical durability, retaining 95.2% of its initial current over 42,000 s in acidic electrolyte. More importantly, RRDE analysis revealed high H_2_O_2_ selectivity exceeding 70%, indicating that the catalyst preferentially promotes the two-electron oxygen reduction pathway. Despite efficient peroxide generation, Co–N@CT–900 maintained superior stability compared to reference catalysts, highlighting its resistance to oxidative degradation. The superior performance arises from the synergistic integration of Co-supported active sites and protective graphitic carbon shells, which enable efficient H_2_O_2_ production while preserving structural integrity under acidic conditions. These findings position Co–N@CT–900 as a promising high-durability catalyst for advanced water treatment technologies based on in situ H_2_O_2_ generation.

## Graphical abstract

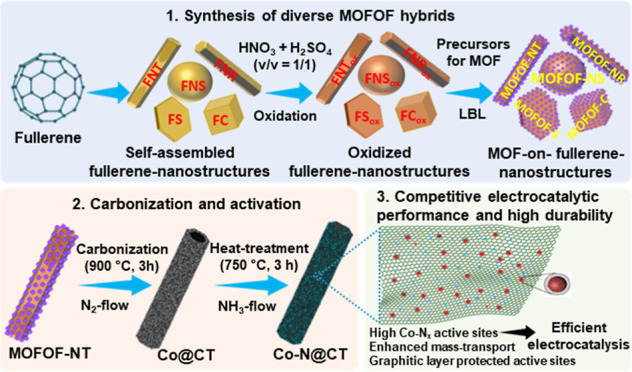

## 1 Introduction

Nanoarchitectonics has emerged as a transformative materials-design philosophy in which molecular units, nanoscale building blocks, and hierarchical architectures are integrated through orchestrated chemical and physical interactions to generate functional materials with precisely defined geometry, composition, and interfaces.^1–3^ Unlike conventional synthesis approaches, nanoarchitectonics enables deliberate spatial organization and electronic coupling of active sites, allowing materials to be engineered from the atomic scale upward to optimize performance.

Within this emerging paradigm, metal–organic framework–on–fullerene (MOFOF) nanoarchitectures^4–7^ represent a fundamentally new class of hybrid structures offering unprecedented control over nanoscale arrangement. Fullerenes such as C_60_ and C_70_ can self-assemble into structurally diverse morphologies—including nanotubes, spheres, rods, sheets, and cubes—each with distinct curvature, porosity, and electronic character.^8–11^ These shape-defined fullerene templates provide programmable environments for directing MOF nucleation and growth, enabling the construction of hybrid architectures in which metal–ligand coordination, crystallization pathways, and interfacial bonding are explicitly governed by the underlying fullerene morphology. In contrast to traditional MOF-based composites,^12–15^ MOFOF systems offer morphology-encoded control over metal dispersion, enhanced thermal robustness, and the potential for producing highly graphitized carbon while maintaining structural integrity during pyrolysis.^16,17^

The transformation of such MOFOF assemblies into advanced carbon materials further amplifies the novelty of this platform. Although MOF-derived carbons are widely valued for their high surface area, nitrogen incorporation, and uniform metal distribution,^18–22^ conventional MOF pyrolysis frequently leads to structural collapse, uncontrolled metal aggregation, and limited graphitization—factors that are especially detrimental for electrocatalysis in acidic media.^23–26^ By contrast, fullerene-guided nanoarchitectonics enables the formation of robust carbon frameworks in which metal species can be spatially confined and encapsulated within graphitic shells. This level of structural precision cannot be achieved through traditional MOF pyrolysis routes.^3,27–29^ Such controlled embedding is particularly transformative for nanostructured carbon–supported nanocatalysts, as it provides simultaneous stabilization of active sites, prevention of dissolution and agglomeration, and significantly improved electron transport. As a result, the nanoarchitectonics-driven MOFOF strategy delivers a new class of carbon nano catalysts with inherently superior durability and structural integrity under harsh reaction conditions, highlighting the conceptual and practical advance enabled by this approach.

In this study, we report a systematic nanoarchitectonic strategy to construct MOFOF materials via a sequential layer-by-layer (LBL) assembly of ZIF-67 onto five distinct self-assembled fullerene morphologies: nanospheres (FNS), nanotubes (FNT), nanorods (FNR), nanosheets (FS), and nanocubes (FC) (Scheme 1). Among these, quasi-1D hollow FNTs offered the most favorable geometry for bidirectional ZIF-67 growth due to their high surface-to-volume ratio and accessible internal channels. Subsequent carbonization of the selected MOFOF-NT at 800–1100 °C followed by nitrogen doping under NH_3_ yielded a series of Co–N@CT nanocomposites featuring uniformly dispersed cobalt nanoparticles encapsulated within graphitic carbon shells (Scheme 1).

**Scheme 1:**
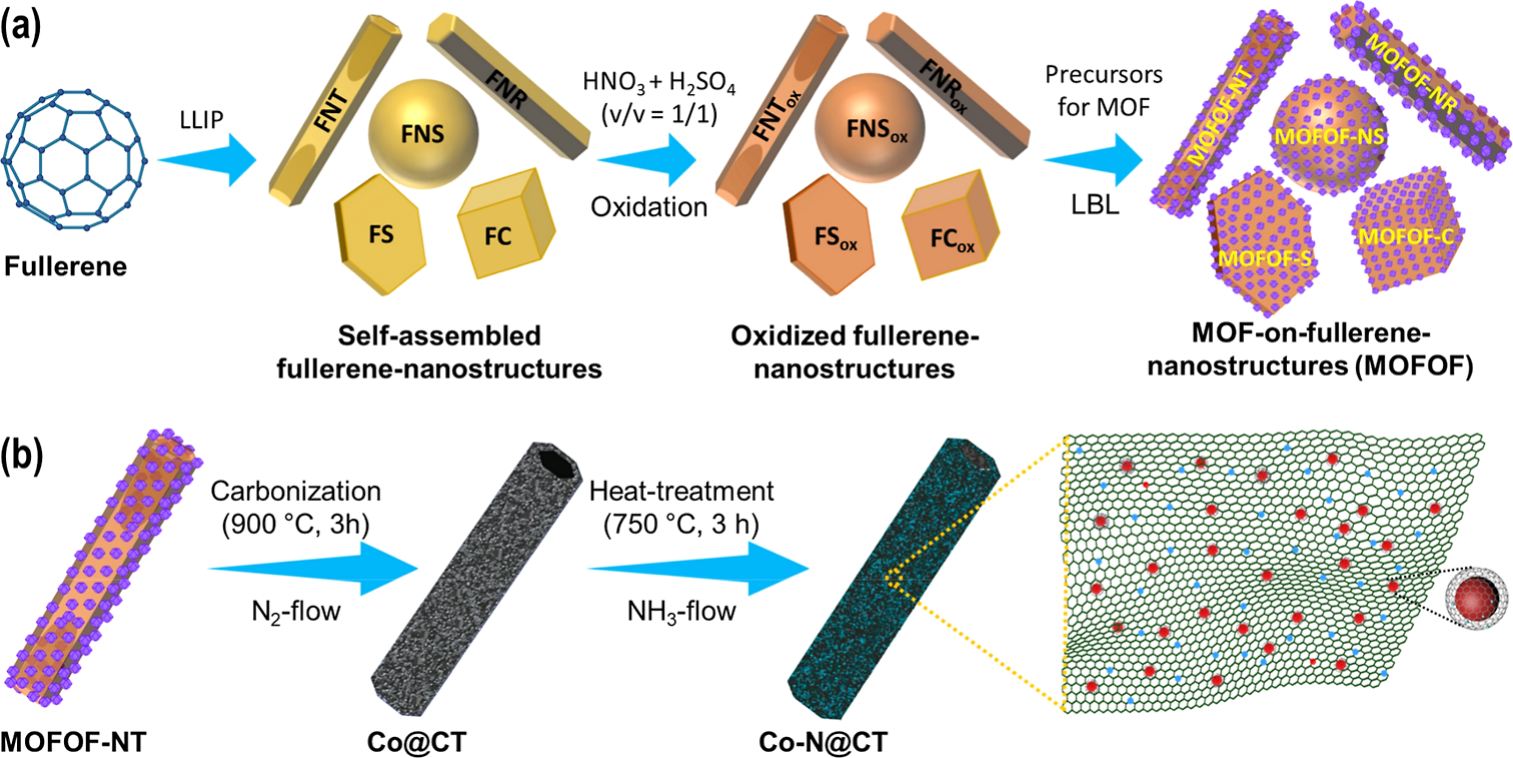
(a) Schematics Representation of the Synthesis of MOFOF Nanostructures and (b) the Conversion of MOFOF-NT (a Representative MOFOF) to N-Doped Co/carbon Nanocomposite

The resulting Co–N@CT-900 catalyst demonstrated outstanding ORR activity and exceptional durability in acidic media, illustrating the effectiveness of the MOFOF nanoarchitectonic approach. More broadly, these results validate MOFOF nanoarchitectonics as a powerful and versatile strategy for engineering next-generation nonprecious metal-based hybrid electrocatalysts, where active sites are spatially organized, structurally protected, and electronically integrated within advanced graphitic carbon frameworks.

## 2 Results and Discussion

### 2.1 Synthesis and Morphological Evolution of MOFOF Nanostructures

The family of MOFOF hybrids, ranging from 0D to 3D, including a quasi-1D hollow nanostructure, were successfully synthesized by growing MOF (ZIF-67) crystals on self-assembled fullerene scaffolds. The fullerene scaffolds exhibited diverse morphologies including FNS (0D), FNR (1D), FS (2D), FC (3D), and FNT (quasi-1D). These architectures were generated using a straightforward three-step protocol (Scheme 1a): (i) shape-controlled synthesis of fullerene assemblies via a liquid–liquid interfacial precipitation technique;^30^ (ii) oxidative treatment using an HNO_3_/H_2_SO_4_ (1:1 v/v) mixture to introduce oxygen-rich functional groups;^31^ and (iii) sequential LBL^32^ deposition of ZIF-67 crystals to form uniform MOF coatings on the oxidized fullerene surfaces.

Figures 1a,d,g,j,m, and S1a, S2a,b, S3a,b, S4a,b, and S5a present the SEM images, at various magnifications where needed, that confirmed the successful formation of fullerene assemblies with diverse morphologies: FNS, FNR, FS, FC, and FNT, respectively.

**Figure 1:**
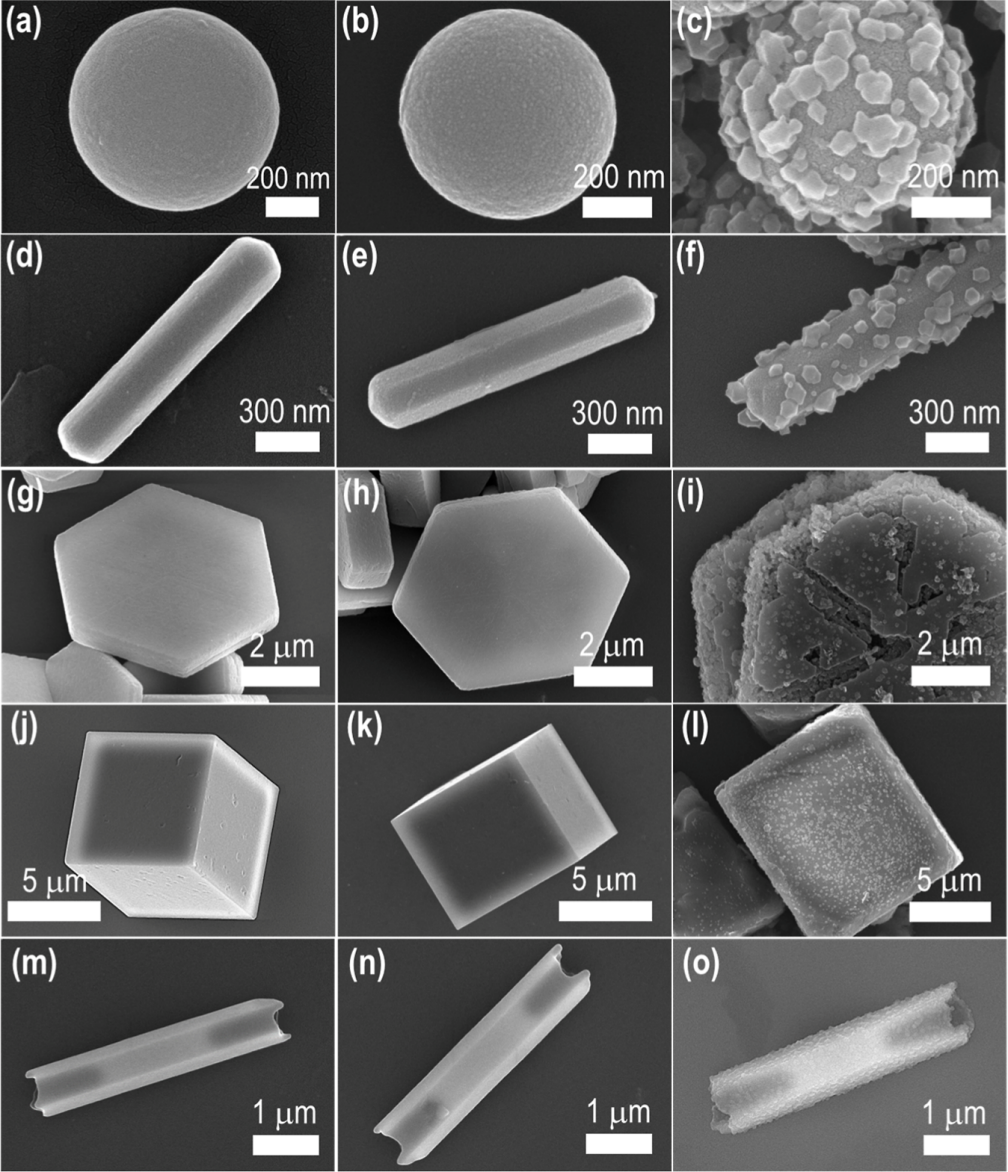
SEM images of various fullerene-based nanostructures and their corresponding modifications (acid treatment and integration of MOF crystals). Pristine fullerene nanostructures include: (a) 0D FNS, (d) 1D FNR, (g) 2D FS, (j) 3D FC, and (m) quasi-1D FNT. Panels (b, e, h, k, and n) show the respective nanostructures after acid treatment or surface oxidation. Panels (c, f, i, l, and o) display the corresponding MOF-on-fullerene (MOFOF) composites.

Size and shape distribution analyses (Figures S1b, S2c,d S3c,d S4c and S5b,c) revealed consistent dimensions: FNS (529 ± 2 nm diameter), FNR (2.22 ± 0.02 μm length; 270 ± 0.5 nm diameter), FS (5.20 ± 0.02 μm size; 428 ± 7 nm thickness), FC (7.34 ± 0.04 μm size), and FNT (3.43 ± 0.03 μm length; 376 ± 7 nm diameter). SEM images of the oxidized samples (Figures 1b,e,h,k,n, and S1c, S2e,f, S3e,f, S4d,e and S5d) and their respective histograms (Figures S1d, S2g,h, S3g,h, S4f, and S5e,f) confirmed that acid treatment preserved morphology, particle dimensions, and surface textures even after oxidation under strong acid conditions. This stability highlights the robustness of fullerene assemblies, even under harsh oxidative conditions.

Surface property modifications were further validated by contact angle analysis (Figure S6a), which showed increased hydrophilicity postoxidation. FTIR spectra (Figure S6b) revealed the presence of oxygen-containing functional groups, with distinct vibrational bands at 1035 cm^−1^ (C–O), 1360 cm^−1^ (C–OH), and 1635 cm^−1^ (C=O), confirming successful functionalization.^33^ Zeta potential measurements (Figure S7) indicated enhanced surface negativity across all oxidized fullerene morphologies, attributed to the formation of oxygen-containing functional groups,^34^ observed in FTIR analysis. Collectively, these analyses confirm the effective surface modification of fullerene nanostructures to facilitate successful MOF nucleation.

To achieve uniform MOF crystal deposition on fullerene nanostructures, a sequential LBL growth strategy was employed by alternating exposure of the oxidized fullerene nanostructures to Co^2+^ and 2-methylimidazole solutions, exploiting electrostatic interactions and coordination chemistry to ensure uniform nucleation and minimize unwanted agglomeration. Typically, oxidized fullerene nanostructures—bearing negatively charged surfaces—were first exposed to a Co^2+^ solution, which promoted the electrostatic binding of metal ions to the surface. This was followed by the addition of the ligand solution, enabling coordination with the adsorbed Co^2+^, promoting localized and controlled nucleation of ZIF-67. The washing of the unbound metal ions and ligands prevents uncontrolled surface nucleation. SEM analyses of the resulting MOFOF hybrids (Figures S8–S12) confirmed dense, conformal ZIF-67 growth on each fullerene scaffold, with crystal sizes consistently in the 75–78 nm range. Notably, the FNT-based hybrid (MOFOF-NT) demonstrated ZIF-67 deposition on both outer and inner surfaces, a feature enabled by its hollow quasi-1D architecture and high surface area.

XRD patterns of MOFOFs (Figure S13) exhibited characteristic diffraction peaks of ZIF-67 at 2θ = 7.28°, 10.34°, 12.65°, and 17.93°, corresponding to the (110), (200), (211), and (222) planes across all morphologies.^35^ Differences in relative peak intensities suggested subtle variations in MOF crystallinity among the MOFOF types. Further comparisons among ZIF-67, FNT_ox_, and MOFOF-NT (Figure S14a) revealed a combination of diffraction features from both the fullerene and MOF components. For FNT_ox_, prominent peaks at 2θ = 11.36°, 19.31°, and 20.81° were attributed to the (210), (401), and (410) planes of fullerene or oxidized fullerenes.^36^ The MOFOF-NT hybrid retained these features along with ZIF-67 peaks, albeit with minor shifts—indicative of successful hybrid formation via chemical integration. Complementary FTIR analysis (Figure S14b) further supported the coexistence of both components. MOFOF-NT exhibited additional bands at 1562 and 1305 cm^−1^, corresponding to C=N and C–C stretching vibrations from the imidazole ligand in ZIF-67.^37^ Raman spectra (Figure S14c) of FNT_ox_, indicating that oxidation preserved the π-conjugated structure of fullerenes^38^ and the appearance of a peak at 688 cm^−1^, attributed to Co–N vibrations for the MOFOF-NT, further confirmed the successful integration of cobalt coordination sites into the MOFOF-NT hybrid.^39^

Among all the synthesized MOFOF nanocomposites, the quasi-1D MOFOF-NT architecture exhibited the most advantageous structural attributes that provide a high surface-to-volume ratio, which facilitates greater exposure of active sites compared to 0D, 2D, and 3D structures. Its hollow tubular morphology offers accessible internal channels. In addition, the quasi-1D structure enables uniform and both side growth of the ZIF-67 precursor on the FNT surface, resulting in a homogeneous spatial distribution of Co species. Upon pyrolysis, this leads to the formation of well-dispersed, stable active sites, which are critical for downstream applications. These combined structural and electronic advantages of quasi-1D dimensionalities were chosen as the optimal precursor for fabricating nitrogen-doped Co–carbon electrocatalysts via thermal treatment. Its morphology and structural robustness rendered it an ideal platform for deriving functional materials with tailored catalytic properties.

### 2.2 Structural and Surface Characterization of Carbonized Samples

The MOFOF nanoarchitectonics strategy, the integration of ZIF-67 onto fullerene nanotube (FNT) scaffolds is a single crystal and enables a uniform spatial distribution throughout the surface. During pyrolysis, the framework evolves into a hierarchical dual carbon architecture, where Co species are uniformly distributed and encapsulated within graphitized carbon and embedded in a porous carbon matrix. The preparation of nitrogen-doped cobalt–carbon nanocomposites (Co–N@CT) was achieved through a two-step thermal treatment protocol (Scheme 1b). In the initial step, the MOFOF-NT hybrid precursor underwent carbonization under an inert nitrogen atmosphere at various temperatures, exceeding the decomposition threshold of fullerene. The resulting carbonized intermediates were then subjected to acid leaching with HCl to remove unstable or unencapsulated cobalt species and denoted as Co@CT–x (where “x” denotes the carbonization temperature). The second step involved ammonolysis under continuous NH_3_ flow at 750 °C for 3 h to introduce nitrogen functionalities and resulting materials designated as Co–N@CT–x.

Thermogravimetric analysis (TGA, Figure S15) was conducted to understand the thermal decomposition behavior of the individual components (FNT_ox_, ZIF-67) and the composite (MOFOF-NT), which guided the carbonization temperature selection. FNT_ox_ exhibited thermal degradation starting around 700 °C, completing near 900 °C, while ZIF-67 underwent rapid decomposition at approximately 425 °C, consistent with prior reports.^40^ Interestingly, the composite MOFOF-NT displayed a shifted decomposition pattern, with ZIF-67 degrading at a higher temperature (∼550 °C), indicative of mutual thermal stabilization between the ZIF-67 and fullerene matrix. This informed the selection of 800–1100 °C as the carbonization range.

Figure S16 presents high-magnification SEM images of both Co@CT–x and Co–N@CT–x materials. The Co@CT–x series (Figure S16a–d) displays rough, porous carbon architectures characterized by embedded, shrunken ZIF-67 residues and sporadically aggregated metallic cobalt nanoparticles. A clear morphological evolution is observed with increasing pyrolysis temperature, where Co@CT–900 and Co@CT–1000 exhibit more homogeneous particle dispersion and interconnected carbon frameworks. After ammonia treatment, the resulting Co–N@CT–x samples (Figure S16e–h) preserve the overall porous morphology but exhibit enhanced surface texturing and subtle structural reorganization, likely due to nitrogen incorporation and micropore formation induced by high-temperature NH_3_ treatment.

The structural and chemical transformations were confirmed through comprehensive XRD, Raman, XPS, and nitrogen sorption analyses (Figures S17 and S18). XRD patterns (Figure S17a) show the disappearance of characteristic peaks from MOFOF-NT, confirming successful carbonization across all pyrolysis temperatures. The presence of broad reflections at ∼2θ = 26° and 44° corresponded to the (002) and (101) planes of graphitic carbon, indicative of partial graphitization that increases with increasing temperature. Additionally, peaks at 2θ ≈ 44.2° and 51.5° were assigned to the (111) and (200) planes of metallic cobalt (Co^0^, JCPDS no. 15–0806).^41^ In the Co–N@CT–x series (Figure S18a), the NH_3_ treatment induces slight reorganization of the metal species and nitrogen incorporation, leading to marginally enhanced crystallinity.

Raman spectra (Figures S16b and S17b) reveal two characteristic bands at ∼1350 cm^−1^ (D-band) and ∼1580 cm^−1^ (G-band), corresponding to disordered carbon and graphitic domains, respectively. The intensity ratio (*I*_G_/*I*_D_) indicates increasing graphitization with higher pyrolysis temperatures. The trends remain similar for both the Co@CT–x series and Co–N@CT–x series with a slight increase after the ammonolysis. Notably, Co–N@CT–900 presents the most balanced structure, featuring metallic cobalt, graphitic carbon, amorphous domains, and moderate *I*_G_/*I*_D_ ratio. This combination implies a favorable balance between graphitization and defect sites which is often considered ideal for effective catalytic site dispersion in electrocatalysis.^42,43^

BET surface area and pore structure analyses (Figures S17c–e, S18c–e, and Table S1) reveal that all samples possess hierarchical porosity with dominant microporous contributions. Ammonia treatment induces controlled etching of the carbon matrix, leading to enhanced porosity and improved accessibility of active sites. As a result, the Co–N@CT–x samples retain their overall morphology while exhibiting increased surface texturing and micropore development after ammonolysis. This is supported by BET analysis, which shows a significant increase in surface area and micropore volume after NH_3_ treatment. Notably, Co–N@CT–900 exhibits a high surface area (1260 m^2^ g^−1^) and microporous surface area (1079 m^2^ g^−1^), while maintaining a consistent average pore diameter (∼3.7–3.9 nm) (Table S1). The combined effects of enhanced nitrogen doping and hierarchical porosity contribute to improved mass transport and greater exposure of catalytically active sites. XPS survey scans of Co@CT–x (Figure S17f) and Co–N@CT–x (Figure S18f) confirms the presence of C, N, O, and Co in all samples, with elemental compositions summarized in Table S2. The cobalt content gradually decreases with rising the pyrolysis temperature, likely due to enhanced aggregation of metallic Co at higher temperatures, followed by leaching of metallic Co (uncovered or aggregated) during acid washing, resulting in lower residual Co content. Similarly, nitrogen content in Co@CT–x shows a general downward trend with increasing pyrolysis temperature. However, following ammonolysis of the Co@CT–x, the resulting Co–N@CT–x samples exhibit stable or slightly elevated nitrogen content (Table S2), suggesting effective nitrogen incorporation and potential structural reorganization during NH_3_ treatment.

High-resolution C 1s spectra (Figures S17g and S18g) reveal a dominant peak at ∼284.7 eV (sp^2^ C=C), with minor contributions from C–N (∼285.8 eV) and C=O (∼289.2 eV). The Co 2p spectra (Figures S17h and S18h) exhibit characteristic Co 2p_3/2_ peaks at 778.5 and 781.6 eV, assigned to metallic Co and Co–N species, respectively, along with a satellite peak near 785.5 eV.^44,45^ The presence of both signals suggests cobalt exists in dual forms: metallic nanoparticles and Co species coordinated with nitrogen in the carbon matrix (Co–N_*x*_). Deconvoluted N 1s spectra (Figures S17i and S18i) identify five distinct nitrogen species: pyridinic-N (N-6, 398.5 eV), Co−nitrogen coordinated species (Co–N_*x*_, 399.1 eV), pyrrolic-N (N-5, 399.5 eV), graphitic-N (N-Q, 402.1 eV), and oxidized nitrogen (N–O_*x*_, 404.1 eV).^46^ The relative abundance of each nitrogen type varies among samples and is summarized in Table S3. These nitrogen functionalities are known to play key roles in tuning electronic structure and catalytic activity in carbon-based materials.^47–50^

To benchmark the advantages of using MOFOF-NT as a precursor for fabricating N-doped Co–carbon nanocomposites, two reference materials, a Co-anchored N-doped composite and a metal-free N-doped carbon, were synthesized under identical two-step thermal protocols (Scheme 1b) using (i) Co^2+^-impregnated FNT_ox_ to yield Co–N_CT–R, and (ii) pristine FNT_ox_ alone to yield N_CT–R. The resulting materials were thoroughly characterized using SEM, TEM, and XPS, and their properties were compared to the representative sample Co–N@CT–900.

SEM images (Figure S19) revealed all three (Co–N@CT–900, Co–N_CT–R, and N_CT–R) retained tubular morphologies with a highly porous carbon framework. STEM images and corresponding particle size distribution profiles (Figure S20) for the Co-containing samples (Co–N@CT–900 and Co–N_CT–R) indicated that Co–N@CT–900 featured more uniformly dispersed Co nanoparticles (∼9–10 nm) compared to Co–N_CT–R. Further insights from TEM analyses (Figures 2, S21, and S22) demonstrate that both Co–N@CT–900 and Co–N_CT–R contain Co nanoparticles embedded within the carbon matrix, while such features are absent in the metal-free N_CT–R sample.

**Figure 2:**
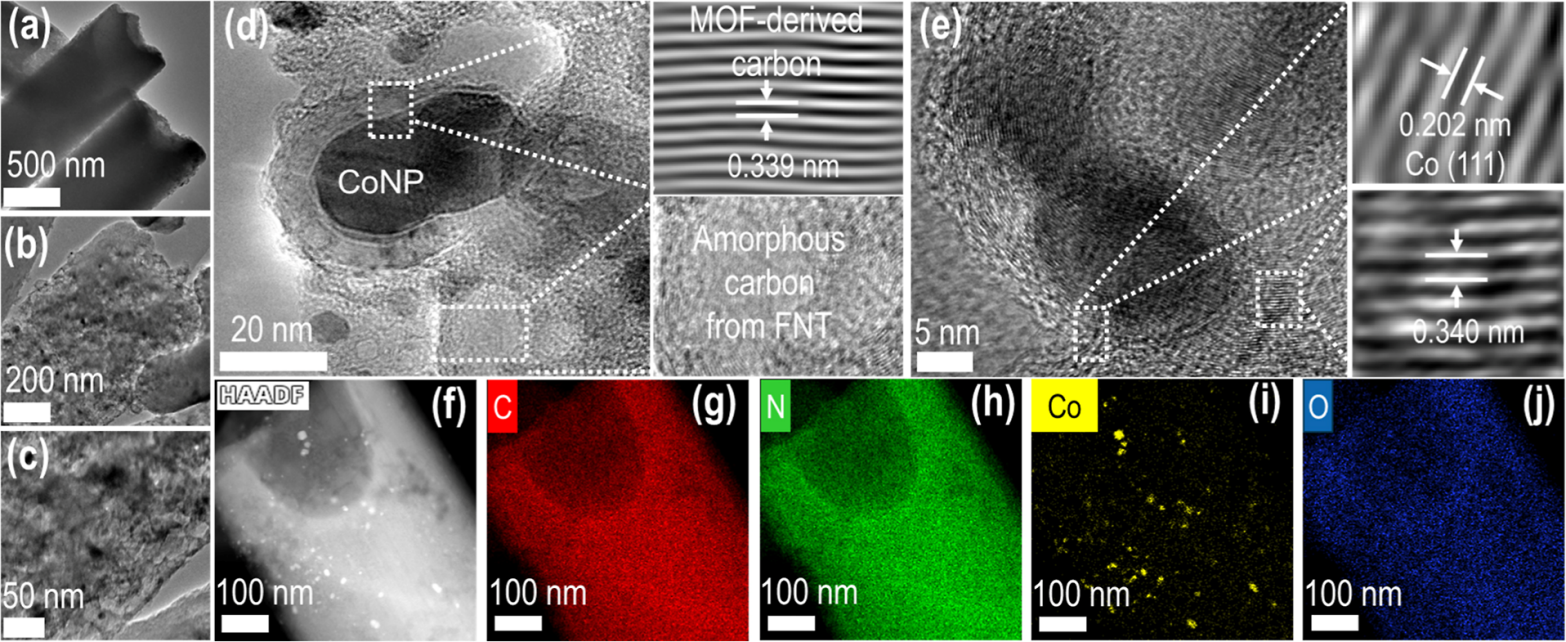
Transmission electron microscopy (TEM) characterization of Co–N@CT–900. (a–c) TEM images at increasing magnifications revealing the porous carbon matrix; (d,e) high-resolution TEM (HRTEM) images showing well-defined lattice fringes corresponding to graphitic carbon and metallic cobalt, indicating crystalline domains. (f) High-angle annular dark-field scanning transmission electron microscopy (HAADF-STEM) image of Co–N@CT–900. (g–j) Corresponding energy-dispersive X-ray (EDX) elemental maps showing uniform spatial distribution of (g) carbon, (h) nitrogen, (i) cobalt, and (j) oxygen within the composite structure.

High-resolution TEM (HR-TEM) images (Figure 2d,e) reveal cobalt nanoparticles encapsulated in multilayered graphitic carbon. Lattice fringes corresponding to the (111) plane of metallic Co (0.202 nm) and graphitic carbon (0.339 nm) confirm this structure.^44,51^ In contrast, HR-TEM images of Co–N_CT–R (Figure S21b–d) display Co nanoparticles embedded in amorphous carbon with limited graphite encapsulation, while the N_CT–R sample (Figure S22b–d) exhibits a smoother carbon surface with no visible metal nanoparticle features. Additionally, HAADF-STEM imaging and elemental mapping (Figures 2f–j and S21f–j) confirm the uniform distribution of Co, N, and O across both Co–N@CT–900 and Co–N_CT–R samples, indicating the formation of well-dispersed active Co and N sites throughout the carbon framework.

Figure 3 illustrates further physicochemical comparisons of the Co–N@CT–900, Co–N_CT–R, and N_CT–R using XRD, Raman, N_2_ sorption, and XPS techniques. The XRD patterns (Figure 3a) of Co–N_CT–R display characteristic diffraction peaks at ∼2θ = 44.2° and 51.5°, similar to those observed for Co–N@CT–900 or Co–N@CTs (Figure S17a) but weaker graphitic carbon features than Co–N@CT–900. The N_CT–R exhibited a broad, featureless pattern indicative of amorphous carbon. Raman spectra (Figure 3b) further confirmed these findings, with Co–N@CT–900 showing a higher degree of graphitization than Co–N_CT–R and N_CT–R. Nitrogen adsorption–desorption isotherms (Figure 3c) and pore size distribution curves (Figure 3d,e) reveal a combination of micropores and mesopores in all samples, with surface area following the trend: Co–N@CT–900 > Co–N_CT–R > N_CT–R.

**Figure 3:**
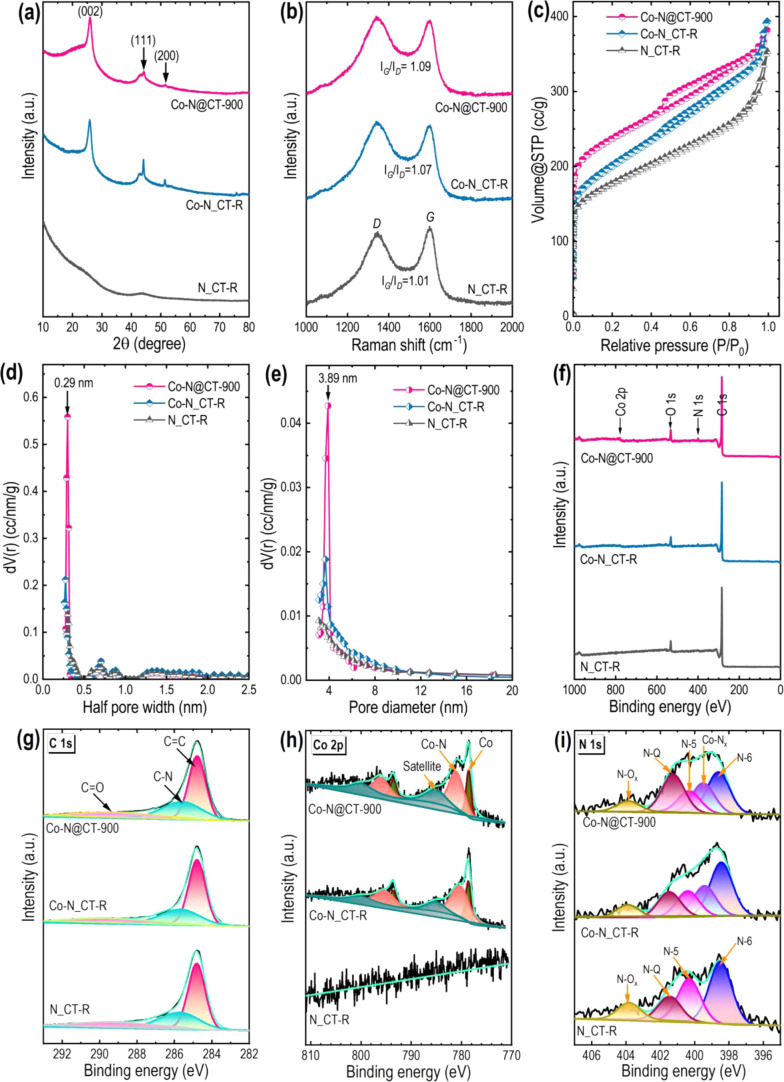
Physiochemical characterization of Co–N@CT–900, Co–N_CT–R, and N_CT–R materials: (a) Powder X-ray diffraction (XRD) patterns; (b) Raman scattering spectra; (c) Nitrogen adsorption–desorption isotherms; (d) Micropore size distribution profiles; (e) Mesopore size distribution profiles; (f) XPS survey spectra; and (g–i) High-resolution XPS spectra of C 1s, Co 2p, and N 1s, respectively.

Figure 3f presents the XPS survey spectra of Co–N@CT–900, Co–N_CT–R, and N_CT–R, highlighting their elemental compositions. The Co–N@CT–900 and Co–N_CT–R samples exhibit comparable contents of C (92.0% vs 93.5%), N (2.45% vs 2.10%), O (4.91% vs 3.78%), and Co (0.63% vs 0.61%). In contrast, N_CT–R contains C (95.2%), N (1.42%), and O (3.42%) but no detectable cobalt. These results indicate metal (Co) assisted nitrogen incorporation, as evidenced by the higher nitrogen content of the Co-containing samples compared to the metal-free N_CT–R. High-resolution C 1s spectra (Figure 3g) for Co–N_CT–R and N_CT–R show a dominant peak at ∼284.7 eV, corresponding to sp^2^-hybridized C=C bonds, along with minor peaks at ∼285.8 eV and ∼289.2 eV, attributed to C–N and C=O groups, that are evident in Co–N@CT–900. The Co 2p spectra (Figure 3h) of Co–N_CT–R display well-defined Co 2p_3_/_2_ peaks at 778.5 and 781.6 eV, corresponding to metallic Co and Co–N_*x*_ species, respectively, accompanied by a satellite peak near 785.5 eV, similar to the Co–N@CT–900. These features suggest the coexistence of cobalt in both metallic Co nanoparticles and Co–N_*x*_ moieties within the carbon matrix. Furthermore, the deconvoluted N 1s spectra (Figure 3i) reveal five nitrogen species: pyridinic-N (N−6, 398.5 eV), Co–N_*x*_ (Co–N_*x*_, 399.1 eV), pyrrolic-N (N−5, 399.5 eV), graphitic-N (N–Q, 402.1 eV), and oxidized-N (N–O_*x*_, 404.1 eV). A comparison of their relative abundances (Table S2) shows that Co–N@CT–900 contains nearly double the N–Q content (0.65%) compared to Co–N_CT–R (0.35%), while N−6 (0.72% vs 0.69%) and Co–N_*x*_ species (0.52% vs 0.41%) are present in comparable proportions. These variations in nitrogen bonding configurations, despite similar overall compositions, likely influence catalytic efficiency and durability by altering local electronic environments and active site structures.

In summary, the MOFOF-NT (selected MOFOF) structure successfully transforms into a hierarchical dual-carbon architecture, in which Co species are dispersed within a defect-rich porous carbon matrix and encapsulated by graphitic carbon layers. These graphitic layers serve as protective shells that effectively inhibit Co aggregation while facilitating efficient electron transport during catalysis, thereby contributing to the overall structural robustness and catalytic performance.

### 2.3 Oxygen Reduction Reaction (ORR) Performance

The electrocatalytic activity of the carbonized (Co@CT–x) and nitrogen-doped (Co–N@CT–x) nanocomposites toward the ORR was systematically assessed using linear sweep voltammetry (LSV) with a rotating ring-disk electrode (RRDE) in O_2_-saturated 0.1 M H_2_SO_4_ electrolyte at 1600 rpm (Figure S23). For the Co@CT–x series (Figure S23a), onset potentials (E_onset, at 0.1 mA cm^−2^) were 0.67, 0.70, 0.68, and 0.65 V (vs RHE) for Co@CT–800, Co@CT–900, Co@CT–1000, and Co@CT–1100, respectively. The results indicate that carbonization at 900 °C yields the most electrochemically active structure among the Co@CT–x series, as higher temperatures negatively impact activity due to possible sintering of active Co species.

Following NH_3_ treatment, all samples in the Co–N@CT–x series exhibited a noticeable enhancement in ORR activity, indicating the crucial impact of nitrogen incorporation. The onset potentials improved significantly in the order: Co–N@CT–900 (0.78 V) > Co–N@CT–1000 (0.76 V) > Co–N@CT–800 (0.74 V) > Co–N@CT–1100 (0.72 V) (Figure S23b). This result highlights the key role of nitrogen doping in modulating the electronic structure around cobalt centers and facilitating more efficient oxygen adsorption and reduction pathways. Although a high surface area generally facilitates in exposing more catalytic sites and improve O_2_ mass transport, all Co–N@CT–x samples in this study possess comparably high surface areas. Therefore, the observed differences in ORR performance are attributed primarily to the differences in the chemical nature and spatial distribution of catalytically active sites, namely, variations in cobalt coordination states and nitrogen functionalities rather than surface area alone. Specifically, the weakest performance of Co–N@CT–1100 corresponds to its minimal Co and N content, suggesting a depletion of active site density at elevated temperatures. Conversely, Co–N@CT–900 exhibits superior ORR activity compared to Co–N@CT–800 despite its slightly lower elemental content, indicating that catalytic performance is primarily governed by optimized electronic structure and favorable Co–N_*x*_ configurations. This is further supported by the potassium thiocyanate poisoning study (Figure S24), where the addition of SCN^−^ ions significantly suppress catalytic activity by selectively binding to Co sites, thereby blocking Co–N_*x*_ active centers and hindering surface charge transfer kinetics.^52–54^

To better understand the ORR performance of Co–N@CT–900, Figure 4a compares its LSV profile with two reference catalysts, Co–N_CT–R and N_CT–R, synthesized under a similar thermal protocol. Co–N@CT–900 demonstrated significantly higher activity, outperforming Co–N_CT–R (0.70 V) and the metal-free N_CT–R (0.54 V). To further elucidate the reaction pathway, RRDE measurements were conducted. The corresponding ring currents (Figure 4b) reached approximately 0.3–0.4 mA cm^−2^ in the kinetically relevant potential region. Based on the disk and ring currents, the H_2_O_2_ production ratio was calculated (Figure 4c), revealing that the Co-supported catalysts exhibited H_2_O_2_ selectivity exceeding 70%, indicating that the reaction predominantly proceeds via a 2e^−^ oxygen reduction pathway. Tafel slope analysis further supports distinct kinetic behavior. Co–N@CT–900 exhibited a Tafel slope of 56.6 mV dec^−1^ (Figure 4d), a value close to the theoretical ∼60 mV dec^−1^ regime. This suggests that the initial electron-transfer step is close to equilibrium and that the rate-determining process occurs in subsequent reaction steps. In contrast, Co–N_CT–R and N_CT–R showed higher Tafel slopes, indicating less favorable reaction kinetics. Electrochemical durability was evaluated through accelerated LSV cycling and chronoamperometric testing. As shown in Figure 4e, after five LSV cycles, the normalized half-wave potential (E_1_/_2_) of Co–N_CT–R decreased to ∼0.97 of its initial value, whereas Co–N@CT–900 remained above 0.995. The change in *E*_1/2_ after 100 CV cycles was negligible (Figure S25) demonstrating excellent structural and electrochemical stability. Chronoamperometric measurements at E_1_/_2_ (Figure 4f) further revealed that, after 40,000 s of continuous operation, the current density of Co–N_CT–R decreased to 84% of its initial value, while Co–N@CT–900 retained ∼95%. The stability observed was comparable to that of Pt/C catalysts under acidic conditions (Figure S26). In addition, Figure S27 presents TEM characterization of Co–N@CT–900 after catalysis. TEM images (Figure S27a–d) at different magnifications show that small Co nanoparticles remain uniformly dispersed within the porous carbon matrix. The HRTEM image (Figure S27e) reveals Co nanoparticles encapsulated by multiple graphitic carbon layers. These observations confirm the retention of morphological and structural integrity after catalysis and demonstrate that graphitic carbon encapsulation effectively suppresses Co nanoparticle aggregation.

**Figure 4:**
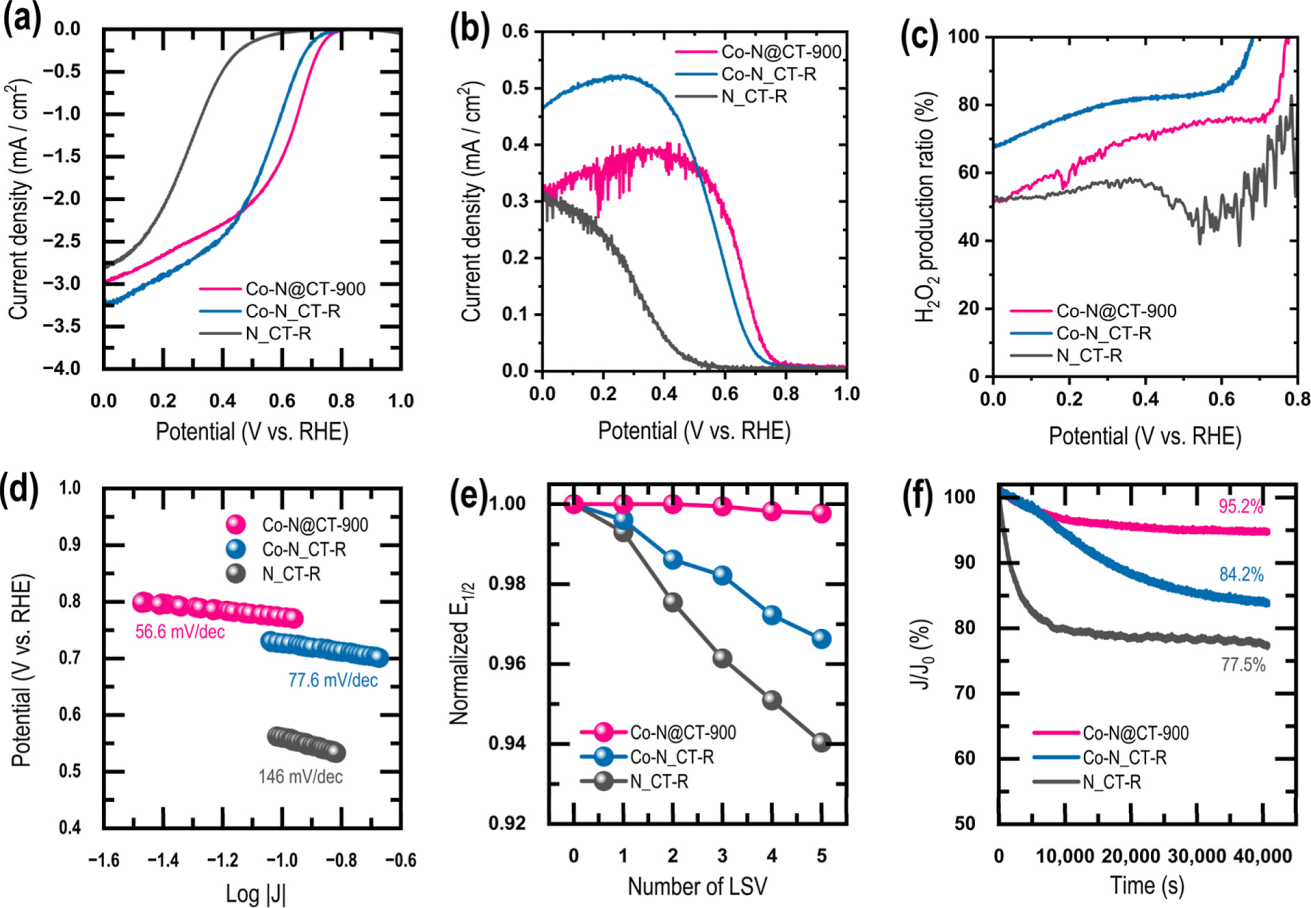
Electrochemical oxygen reduction reaction (ORR) performance of Co–N@CT–900, Co–N_CT–R, and N_CT–R catalysts in O_2_-saturated 0.1 M H_2_SO_4_ electrolyte. (a) Linear sweep voltammetry (LSV) disk currents recorded at 1600 rpm using a rotating ring–disk electrode (RRDE). (b) Corresponding ring currents measured simultaneously during the RRDE experiments. (c) Calculated H_2_O_2_ production ratios derived from disk and ring currents, indicating the selectivity of the ORR pathway. (d) Tafel plots obtained from the kinetic region of the polarization curves. (e) Durability evaluation based on cyclic voltammetry between 1.0 and 0.0 V vs RHE; 20 CV cycles were followed by one LSV measurement, repeated five times, and the normalized half-wave potential (E_1_/_2_) was monitored. (f) Chronoamperometric responses (i–t) showing current retention over prolonged operation.

These results indicate that although the Co-supported catalysts efficiently promote H_2_O_2_ generation, durability—typically a critical limitation—was significantly improved in Co–N@CT–900. This enhanced stability is attributed to the formation of protective graphitic carbon shells that encapsulate the active sites, suppressing oxidative degradation while maintaining catalytic accessibility. Consequently, Co–N@CT–900 represents a catalyst system capable of simultaneously achieving efficient H_2_O_2_ production and high electrochemical durability. Such characteristics highlight its potential as a robust catalyst for advanced water purification technologies based on in situ H_2_O_2_ generation.

## 3 Conclusion

In summary, we have demonstrated a versatile nanoarchitectonics strategy for the controlled assembly of metal–organic frameworks on fullerene templates with diverse dimensionalities (0D–3D), yielding hierarchical nanostructured carbon electrocatalysts. By integrating ZIF-67 onto a series of fullerene nanostructures, including spheres, rods, tubes, sheets and cubes, and subsequently applying high-temperature carbonization with nitrogen doping to a selected MOFOF (quasi-1D FNT), we developed a versatile strategy to derive Co/N-doped carbon materials with tunable architectures and active-site environments. The influential effect of carbonization temperature and importance of treatment under NH_3_ atmosphere was confirmed form the observed Co–N@CT–900 as the most outstanding oxygen reduction performance in acidic media. The Co–N@CT–900 catalyst features a high onset potential, a low Tafel slope, and remarkable durability, retaining 95.2% of its current over 42,000 s. Structural characterization revealed that its superior activity results from the synergistic contributions of dimensionality-tailored morphology, hierarchical porosity, graphitic enrichment, and the stable encapsulation of Co–N_*x*_ active sites within conductive carbon frameworks. This study highlights the versatility of morphology-directed MOFOF nanoarchitectonics across 0D–3D fullerene templates and its promise for creating high-performance, nonprecious metal electrocatalysts for future energy systems.

## 4 Materials And Methods

### 4.1 Materials

Pristine fullerene C_60_ (pC_60_: 99.9%) was purchased from BBS Chemicals, USA. Cobalt nitrate hexahydrate (Co­(NO_3_)_2_·6H_2_O, 99%) and methylimidazole (Mim, 99.8%) were supplied by Tokyo Chemical Industry Co., Ltd., Japan. Isopropyl alcohol (), carbon tetrachloride, *tert*-butyl alcohol, ethanol (99.7%), triethylamine (TEA), mesitylene (99.8%), sulfuric acid (H_2_SO_4_: 98%) and nitric acid (HNO_3_: 65%), were procured from Wako Chemical Corporation, Tokyo, Japan. All other chemical reagents utilized in this study were of analytical grade, and deionized (DI) water was used throughout the experiments.

### 4.2 Method

Experimental details and additional data are supplied in the Supporting Information.

## Supplementary Material



## Notes

Details of synthesis and characterizations such as porosity analysis results, compositions, SEM, contact angle, zeta potential, XRD, FTIR, Raman, TGA, BET, XPS, TEM, LSV, and durability performance analysis results (PDF)

## Abbreviations

FC: fullerene nanocubes
FNR: fullerene nanorods
FNS: fullerene nanospheres
FNT: fullerene nanotubes
FS: fullerene nanosheets
LBL: layer-by-layer
Mim: methylimidazole
MOF: metal organic framework
MOFOF: metal organic framework on fullerene
ORR: oxygen reduction reaction
ZIF-67: zeolite imidazole framework
