# Supplementary material for: Nanoarchitectonics of Metal–Organic Framework on Fullerene Assemblies: Fabrication of Hierarchical Nanostructured Carbon Electrocatalysts
Source: ACS Appl Mater Interfaces. 2026 May 15;18(20):28717–27. doi: 10.1021/acsami.6c03512 (PMC13220222; doi:10.1021/acsami.6c03512)
Supplement: Supplementary file 1 [file am6c03512_si_001.pdf]

Supporting Information

Nanoarchitectonics of Metal–Organic Framework on Fullerene Assemblies: Fabrication of Hierarchical Nanostructured Carbon Electrocatalysts

Rabindra Nath Acharyya[1,2$], Biswa Nath Bhadra[1,3$], Sabina Shahi[1,2], Kenji Hayashida[2,4], Shusaku Fujita[2], Kotaro Takeyasu[5,6], Katsuhiko Ariga[1,7], Lok Kumar Shrestha[1,6*]*

[1]Research Center for Materials Nanoarchitectonics (MANA), National Institute for Materials Science (NIMS), 1-1 Namiki, Tsukuba, Ibaraki 305-0044, Japan

[2]Graduate School of Science and Technology, University of Tsukuba, 1-1-1 Tennodai, Tsukuba, Ibaraki 305-8573, Japan

[3]Institute Charles Gerhardt Montpellier (ICGM), Centre national de la recherche scientifique (CNRS), Montpellier, 34095, France

[4]Graduate School of Environmental Science, Hokkaido University, N10W5, Sapporo, Hokkaido 060-0810, Japan

[5]Institute for Catalysis, Hokkaido University, Kita 21 Nishi 10, Kita-ku, Sapporo, Hokkaido 001-0021, Japan

[6]Department of Materials Science, Institute of Pure and Applied Sciences, University of Tsukuba, 1-1-1 Tennodai, Tsukuba, Ibaraki 305-8573, Japan

[7]Department of Advanced Materials Science, Graduate School of Frontier Sciences, The University of Tokyo, 5-1-5 Kashiwanoha, Kashiwa, Chiba 277-8561, Japan

*Corresponding authors:

ARIGA.Katsuhiko@nims.go.jp (K. Ariga)

SHRESTHA.Lokkumar@nims.go.jp (L.K. Shrestha)

$R.N.A. and B.N.B. contributed equally to this work.

1. Synthesis Methods

1.1 Synthesis of Fullerene Self-Assemblies

Fullerene nanostructures, including nanospheres (FNS), nanorods (FNR), nanosheets (FS), nanocubes (FC), and nanotubes (FNT), were synthesized using the liquid–liquid interfacial precipitation (LLIP) method,[1–9] with minor modifications where necessary. LLIP relies on interfacial nucleation and spontaneous self-assembly of fullerene molecules at the interface between a nonpolar (good) solvent (e.g., mesitylene, *m*-xylene, or carbon tetrachloride, CCl_4) and a polar (poor) solvent (e.g., isopropyl alcohol (IPA) or *tert*-butyl alcohol).

In a typical procedure, fullerene (C_{60} or C_{70}) was first dissolved in a good solvent under sonication and subsequently brought into contact with a poor solvent, triggering interfacial precipitation and self-assembly into distinct nanostructures. The resulting morphology was governed by solvent polarity, mixing conditions, intermolecular interactions, and crystallization parameters. The synthesis procedures for each fullerene nanostructure are described below.

Synthesis of Fullerene Nanorods (FNR). The FNR was prepared following a reported LLIP method[1] with slight modifications to enable scale-up. Briefly, a freshly prepared C_{60} solution in *m*-xylene (50.0 mL, 2 mg.mL^{-1}), obtained by sonication for 1 h followed by filtration, was transferred into a glass vial. Isopropyl alcohol (50.0 mL) was then rapidly added to the solution (solvent ratio 1:1, v/v). After vortex mixing for 30 s, the mixture was incubated at 25 °C for 6 h to allow the desired FNR formation. The resulting precipitate was collected by

centrifugation, washed three times with IPA, and dried overnight at 80 °C under reduced pressure.

Synthesis of Fullerene Sheets (FS). The FS was synthesized following a previously reported method.[5] A saturated solution of C_{60} in CCl_4 was first prepared by dispersing excess C_{60} in CCl_4 (50 mL) under sonication for 1 h, followed by filtration to remove undissolved solids. A 1 mL aliquot of the saturated solution was transferred to a vial and pre-cooled at 5 °C for 20 min. Subsequently, 1 mL of isopropyl alcohol (IPA) was slowly added to initiate controlled crystallization. After gentle hand shaking for 10 s, the mixture was incubated at 5 °C for 24 h. The resulting precipitate was collected by centrifugation and dried overnight at 80 °C under reduced pressure.

Synthesis of Fullerene Cubes (FC). A reported method was followed to synthesize the FC using fullerene C_{70}.[6] A solution of C_{70} in mesitylene (1 mg/mL) was prepared via 1 h sonication and filtration. The C_{70} solution (1 mL) was placed in a vial, and tert-butyl alcohol (5 mL) was added slowly, followed by 30 s vortex mixing. The mixture was kept undisturbed at 25 °C for 24 h to allow crystal growth. The precipitate was separated by centrifugation and dried under reduced-pressure at 80 °C overnight.

Synthesis of Fullerene nanotubes (FNT). A previously reported dynamic LLIP method was employed for the synthesis of FNT, with slight modifications to enable scale-up.[7] A C_{60} solution in mesitylene (1 $mg.mL^{-1}$) was prepared by sonication for 1 h, followed by filtration. IPA (90 mL) was placed in a 150 mL glass vial, after which the freshly prepared C_{60} solution (30 mL) was rapidly added. The resulting mixture was shaken manually for 5 s, vortexed for

1 min, and then incubated at 25 °C for 3 h. The precipitate formed was collected by centrifugation, washed three times with IPA, and dried overnight in a reduced-pressure oven at 80 °C.

Synthesis of Fullerene nanospheres (FNS). Following a reported method,[8,9] FNS was synthesized using a freshly prepared fullerene C_{60} solution in mesitylene (1.0 mg mL^{-1}). Ethylenediamine (EDA, 0.55 mL) was first mixed with mesitylene (5 mL) in a 13.5 mL vial and sonicated for 30 min to obtain a homogeneous solution. Subsequently, the C_{60} solution (5 mL) was gently added, followed by vortex mixing for 30 s. The mixture was kept at 25 °C for 1 h to allow nanosphere formation. The precipitate was collected via centrifugation, washed three times with IPA (3 × 5 mL), and dried under reduced-pressure at 80 °C overnight.

1.2 Surface modification and functionalization of fullerene self-assemblies

Surface functionalization of the fullerene self-assemblies (FNS, FNR, FNT, FS, and FC) was performed under mild conditions to introduce oxygen-containing functional groups while preserving their morphological integrity. Typically, each fullerene assembly (200 mg) was dispersed in a mixed acid solution (20 mL, H_2SO_4:HNO_3 = 1:1, v/v) and subjected to mild sonication for 5 min, followed by static immersion for 1 h at ambient conditions. The oxidized products were collected by centrifugation, thoroughly washed with deionized water until a neutral pH (~7) was reached, and dried overnight in a reduced-pressure oven at 80 °C. The resulting oxidized assemblies (FNS_{ox}, FNR_{ox}, FS_{ox}, FC_{ox}, and FNT_{ox}) were subsequently used as substrates for the fabrication of MOFOF composites.

1.3 Synthesis of MOFOF composites

MOFOF composites were fabricated via a sequential layer-by-layer growth of ZIF-67 (a representative methylimidazole-based MOF) on oxidized fullerene assemblies under ultrasonication. Ultrasonication was deliberately employed to accelerate precursor diffusion, enhance surface coordination, and maintain dispersion stability during each growth cycle. Aqueous solutions of cobalt nitrate hexahydrate ($Co(NO_3)_2{\cdot}6H_2O$, 1 mM, 10 mL) and a ligand mixture containing 2-methylimidazole (Mim) and triethylamine (TEA) (8 mM each, 10 mL) were prepared in separate vials. The oxidized fullerene assembly (50 mg) was first dispersed in deionized water (50 mg mL^{-1}) in a 13.5 mL vial to form a homogeneous slurry. The Co^{2+} solution (1.5 mL) was then added to the dispersion, followed by ultrasonication for 10 min with intermittent manual shaking (5 s after every 2 min of sonication). The resulting mixture was centrifuged, and the supernatant containing uncoordinated Co^{2+} ions was decanted, followed by thorough washing with deionized water to ensure complete removal of unbound metal ions. Subsequently, the ligand solution (Mim + TEA; 1.5 mL) was rapidly introduced to the collected residue, and the same sonication–shaking protocol was repeated for an additional 10 min. After centrifugation and water washing, this growth cycle was repeated four times. The final precipitate was dried overnight in a reduced-pressure oven at 80 °C. This procedure was identically applied to all oxidized fullerene morphologies (FNS_{ox}, FNR_{ox}, FS_{ox}, FC_{ox}, and FNT_{ox}) to obtain the corresponding MOFOF composites.

1.4 Carbonization of MOFOF-NT

A selected MOFOF sample, the MOFOF-NT, was carbonized to produce Co-based hybrid carbon electrocatalysts. The MOFOF-NT composite was subjected to carbonization at four different temperatures (800, 900, 1000, and 1100 °C) under a continuous nitrogen flow (150

cm^3·min^{-1}) in a tube furnace. The temperature was increased at a controlled ramp rate of 5 °C min⁻1, maintained at the target temperature for 3 h, and then allowed to cool to room temperature at a rate of 10 °C min⁻1. After the carbonization complete, the samples were subjected to acid leaching with 1 M HCl for 1 h to remove unstable or undraped cobalt species. The resulting carbonized products were denoted as Co@CT-800, Co@CT-900, Co@CT-1000, and Co@CT-1100.

1.5 NH_3 treatment of Co@CT-x samples

The Co@CT-x materials were subjected to a second thermal treatment under an NH_3 atmosphere to introduce nitrogen functionalities and induce carbon activation. Each carbonized Co@CT-x sample was placed in an alumina boat and treated at 750 °C for 3 h under a continuous NH_3 flow (200 cm^3·min^{-1}). The same temperature ramping conditions were maintained as in the previous carbonization step. After completion of the treatment and cooling to room temperature, the resulting nitrogen-doped carbon composites were denoted as Co-N@CT-800, Co-N@CT-900, Co-N@CT-1000, and Co-N@CT-1100.

1.6 Synthesis of reference catalysts: Co-N_CT-R and N_CT-R

Two reference carbon catalysts were additionally prepared by carbonization followed by NH_3 activation using FNT_{ox} and cobalt-impregnated FNT_{ox}. To impregnate cobalt, an aqueous dispersion of FNT_{ox} (50 mg·mL^{-1}) was prepared, to which a Co^{2+} solution was added. The resulting mixture was sonicated for 10 min with intermittent manual shaking (~5 s every minute) to facilitate impregnation/coordination of cobalt ions onto the FNT_{ox}. After centrifugation to remove the solvent, a freshly prepared $NaBH_4$ solution (1 mM, 10 mL) was

introduced, and the mixture was sonicated again under identical conditions to chemically reduce the impregnated/coordinated cobalt species. The resulting solid was collected by centrifugation, thoroughly washed with deionized water (three times), and dried overnight under reduced pressure at 80 °C. The dried material was then carbonized at 900 °C for 3 h under a nitrogen atmosphere and subsequently treated with NH_3 under the same nitrogen-doping conditions described above, yielding the reference catalyst denoted as Co-N_CT-R. A cobalt-free reference carbon (N_CT-R) was obtained by directly carbonizing the FNT_{ox} at 900 °C for 3 h, followed by NH_3 treatment under identical conditions.

2. Characterizations

The synthesized MOFOF-derived composites were systematically characterized to elucidate their structural, morphological, and chemical features. The morphology and microstructure were examined by scanning electron microscopy (SEM, Hitachi S-4800, Tokyo, Japan) at 10 kV, transmission electron microscopy (TEM, JEM2100F, JEOL, Tokyo, Japan) at 200 kV, and scanning transmission electron microscopy (STEM, Hitachi S-4800) at 30 kV. Thermal stability and composition were assessed via thermogravimetric analysis (TGA, STA 2500, NETZSCH, Germany). Crystallinity and phase composition were analyzed using powder X-ray diffraction (PXRD, Rigaku RINT2000, Tokyo, Japan) with Cu–Kα radiation (λ = 0.1541 nm) at 40 kV. Chemical bonding and functional groups were probed by Fourier-transform infrared spectroscopy (FT-IR, NICOLET iS20, Thermo Fisher Scientific, Waltham, MA, USA) and Raman spectroscopy (NRS-3100, JASCO, Tokyo, Japan). Surface composition and elemental states were determined by X-ray photoelectron spectroscopy

(XPS, Thermo Electron, Karlsruhe, Germany) using monochromatic Al–Kα radiation (photon energy = 15 keV). Textural properties, including surface area and pore size distribution, were evaluated via nitrogen adsorption–desorption isotherms at 77 K using a Quantachrome Autosorb-1 instrument (Boynton Beach, FL, USA).

3. Electrochemical Measurements

Electrochemical performance, including ORR activity, cycle stability, and chronoamperometric response, was evaluated using a Metrohm Autolab Potentiostat-Galvanostat (PGSTAT302N) in a conventional three-electrode setup. A catalyst-coated glassy carbon electrode served as the working electrode, a KCl-saturated Ag/AgCl electrode as the reference, and a platinum wire as the counter electrode.

The glassy carbon electrode (disk) was mechanically polished using alumina (Al_2O_3) slurry and diamond powder, followed by thorough rinsing with ultrapure water to ensure a clean and uniform surface. Catalyst inks were prepared by dispersing 5 mg of each catalyst in a mixture of isopropanol (600 μL), water (360 μL), and Nafion solution (40 μL), followed by 10 min sonication to obtain a homogeneous suspension. Then, 20 μL of catalyst ink was drop-cast onto the glassy carbon disk and allowed to dry at room temperature, resulting in a loading of approximately 0.1 mg per electrode.

All measurements were conducted in 0.1 M H_2SO_4 electrolyte, saturated with either N_2 or O_2. Linear sweep voltammetry (LSV) was performed at a scan rate of 10 mV s^{-1} with the working electrode rotated at 1600 rpm. The ORR current was obtained by subtracting the background current recorded in N_2-saturated electrolyte from the current measured in O_2-saturated conditions ($I_{O_2} - I_{N_2}$). Current densities were normalized to the geometric area of the electrode. A catalyst poisoning test for ORR activity was conducted in a potassium thiocyanate-

containing electrolyte (0.1 M H_2SO_4 + 10 mM KSCN), using the same loading of Co–N@CT–900 and identical measurement conditions. A catalyst poisoning test for the ORR activity was conducted in a potassium thiocyanate-containing electrolyte 0.1 M H_2SO_4 + 10 mM KSCN), using the same loading of Co–N@CT–900 catalyst and identical measurement conditions.

All potentials were initially measured versus Ag/AgCl and converted to the reversible hydrogen electrode (RHE) scale using the equation:

$$E\ (RHE) = E\ (Ag/AgCl) + 0.059 \times pH + 0.199\ V$$

Catalyst durability was evaluated using both cyclic voltammetry (CV) and chronoamperometry. For CV-based stability, the electrolyte was first purged with O_2 for 20 min, and an initial LSV curve was recorded. CV was then performed in the potential range of 1.0–0.0 V vs. RHE for 20 consecutive cycles, followed by a subsequent LSV measurement under identical conditions. This CV–LSV sequence was repeated five times to monitor performance retention over extended cycling, with half-wave potentials ($E_{1/2}$) normalized for comparison. Chronoamperometric measurements were carried out in O_2-saturated 0.1 M H_2SO_4 at the half-wave potential of each catalyst to assess long-term current stability.

Table S1. Specific surface area and textural properties of the materials were obtained from nitrogen adsorption-desorption isotherms, DFT and BJH-method.

Materials	*SSA* (m^2g^{-1})	S_{mic} (m^2g^{-1})	S_{mes} (m^2g^{-1})	V_p (cm^3g^1)	V_{mic} (cm^3g^{-1})	W_p (nm)	D_P (nm)
Co@CT-800	398	287	111	0.45	0.27	0.27	3.66
Co@CT-900	665	474	192	0.69	0.39	0.31	3.71
Co@CT-1000	665	534	129	0.63	0.36	0.29	3.69
Co@CT-1100	248	119	130	0.39	0.20	0.19	3.68
Co-N@CT-800	951	828	123	0.71	0.47	0.30	3.88
Co-N@CT-900	1260	1079	181	0.81	0.54	0.27	3.89
Co-N@CT-1000	1059	862	196	0.77	0.52	0.26	3.68
Co-N@CT-1100	773	601	172	0.65	0.39	0.27	3.68
Co-N_CT-R	109	895	184	0.78	0.54	0.26	3.68
N_CT-R	855	724	130	0.77	0.47	0.30	3.10

SSA = total specific surface area, S_{micro} = surface area of micropore, S_{meso} = surface area of mesopore, V_p = total pore volume, V_{micro} = micropore volume, W_p = Average half pore width obtained from the DFT model, and D_p = Average pore diameter obtained from the BJH analysis.

Table S2. Elemental composition of the synthesized carbon nanocomposites as determined by XPS analysis.

Materials	C (atom%)	N (atom%)	O (atom%)	Co (atom%)
Co@CT-800	90.74	2.28	6.22	0.76
Co@CT-900	92.64	1.93	4.70	0.74
Co@CT-1000	94.35	1.24	3.81	0.60
Co@CT-1100	97.80	0.58	1.41	0.21
Co-N@CT-800	90.66	2.59	6.04	0.71
Co-N@CT-900	92.0	2.45	4.91	0.63
Co-N@CT-1000	93.14	1.79	4.76	0.31
Co-N@CT-1100	97.99	0.68	1.13	0.21
Co-N_CT-R	93.5	2.10	3.78	0.61
N_CT-R	95.2	1.42	3.42	0.00

Table S3. Nitrogen species composition of the studied materials, determined from the integration of deconvoluted N 1s XPS spectra.

Materials	N (atom%)	N-6 (atom%)	N-5 (atom%)	N-Q (atom%)	Co-N_x (atom%)	N-O_x (atom%)
Co@CT-800	2.28	0.4	0.67	0.44	0.51	0.26
Co@CT-900	1.93	0.24	0.59	0.48	0.42	0.2
Co@CT-1000	1.24	0.18	0.33	0.31	0.25	0.17
Co@CT-1100	0.58	0.06	0.16	0.16	0.09	0.11
Co-N@CT-800	2.59	0.92	0.44	0.55	0.45	0.23
Co-N@CT-900	2.45	0.72	0.37	0.65	0.52	0.19
Co-N@CT-1000	1.79	0.49	0.26	0.48	0.32	0.24
Co-N@CT-1100	0.68	0.2	0.08	0.22	0.08	0.1
Co-N_CT-R	2.10	0.7	0.37	0.36	0.41	0.26
N_CT-R	1.42	0.59	0.47	0.18	ND	0.18

ND: Not detected

Table S4: Summary of the previously reported Cobalt-based carbon catalysts in terms of their synthesis and ORR activity in acidic electrolyte.

Catalysts	RRDE activity vs RHE (V)	Half-wave potentials ($E_{1/2}$)	Tafel slope $mVdec^{-1}$	Durability	References
**Co-N@CT-900**	**0.78 0.1 M H_2SO_4**	**0.64**	**56**	**95.2 % 42,000 S, $E_{1/2}$ = 2 mV**	**This work**
Co-N_4	0.82 0.1 M $HClO_4$	0.70	103	85% 36,000 S	10
Co@N–C-700	0.80 0.1 M $HClO_4$	0.65	-	77 % 36,000 S	11
Co-N/C fiber	0.80 0.5 M H_2SO_4	0.68	55	~78 % 40,000 S	12
Co-N/Cs	0.83 0.5 M H_2SO_4	0.70	45	-	13
Co–N SAC	0.78 0.1 M $HClO_4$	-	87	90 % 20,000 S	14
Co-SA-N/C	~0.93 0.5 M H_2SO_4	0.80	-	83.3 % 100 h. $E_{1/2}$ = 30 mV	15
Co/HNCS	~0.84 0.5 M H_2SO_4	0.77	34	$E_{1/2}$ = 3 mV	16

Co–N–C	0.77<br>0.5 M H_2SO_4	0.50	112		17
Co–N–C	0.78<br>0.1 M $HClO_4$	0.48	-	-	18
FeCo-NC	0.86<br>0.1 M $HClO_4$	-	65	91.3 %<br>25,000 S	19
Co–N–GA	0.88<br>0.5 M H_2SO_4	0.73	-	130 mV<br>20 mV	20
Co–N–C	~0.84<br>0.1 M $HClO_4$	0.76	93	$E_{1/2}$ = 4 mV	21
C–N–Co	~0.88<br>0.5 M H_2SO_4	0.79	-	9 mV	22
Pt/C	~0.94<br>0.5 M H_2SO_4	0.82	-	64.7 %<br>60,000 S	23
Pt/C	~0.92<br>0.1 M $HClO_4$	0.804	69	~34 %<br>40,000 S	24

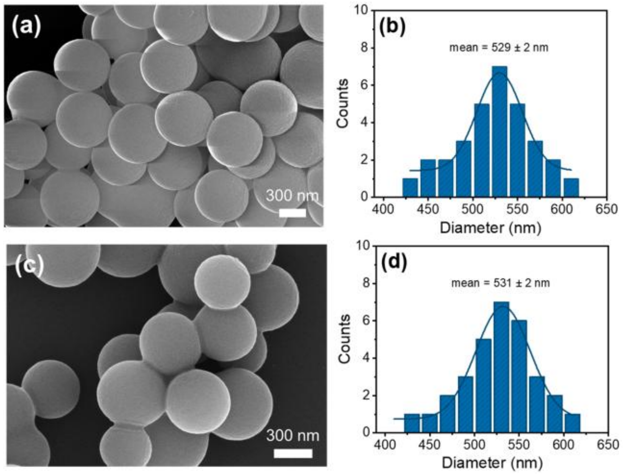

Figure S1. Scanning electron microscopy (SEM) images and corresponding diameter distribution histograms of (a, b) pristine fullerene nanospheres (FNS) and (c, d) oxidized fullerene nanospheres (FNS_{ox}).

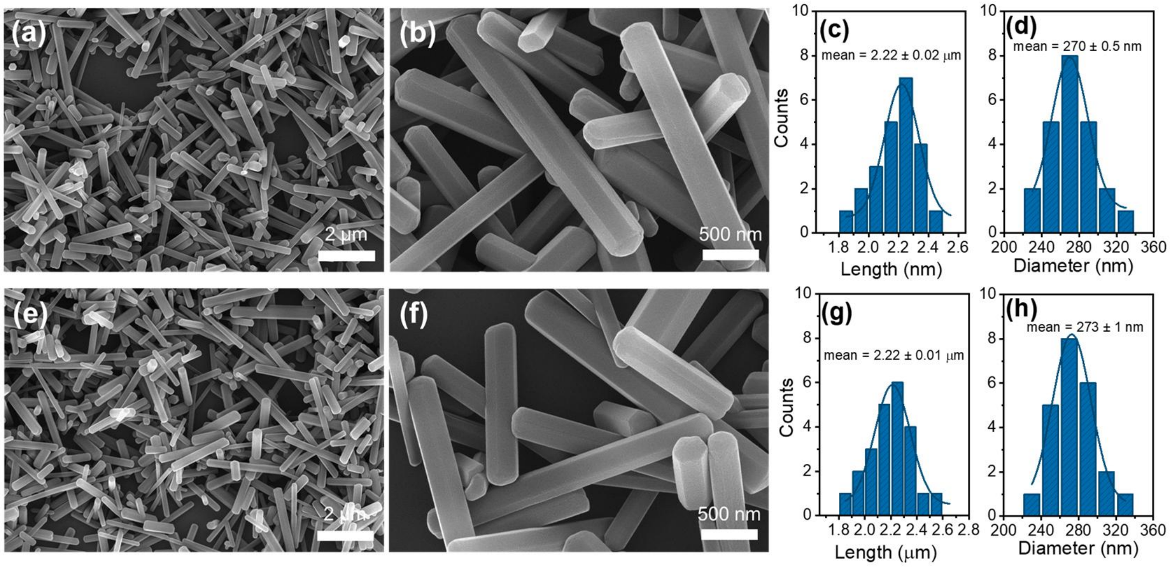

Figure S2. Scanning electron microscopy (SEM) images and corresponding histograms showing the length and diameter distributions of (a–d) pristine fullerene nanorods (FNR) and (e–h) oxidized fullerene nanorods (FNR_{ox}).

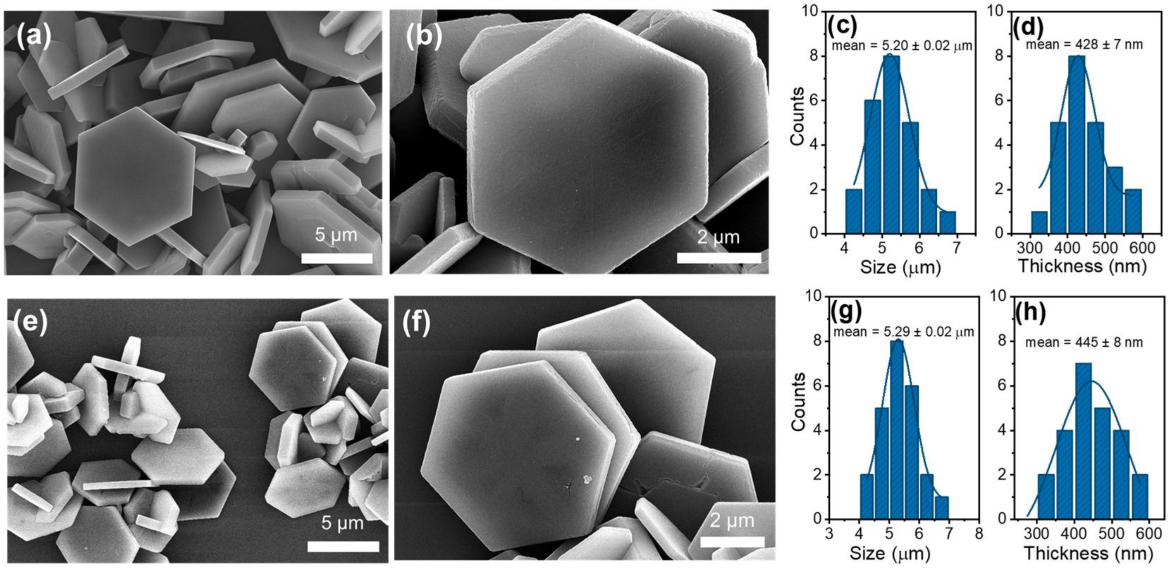

Figure S3. Scanning electron microscopy (SEM) images and corresponding histograms showing the width and height distributions of (a–d) pristine fullerene sheets (FS) and (e–h) oxidized fullerene sheets (FS_{ox}).

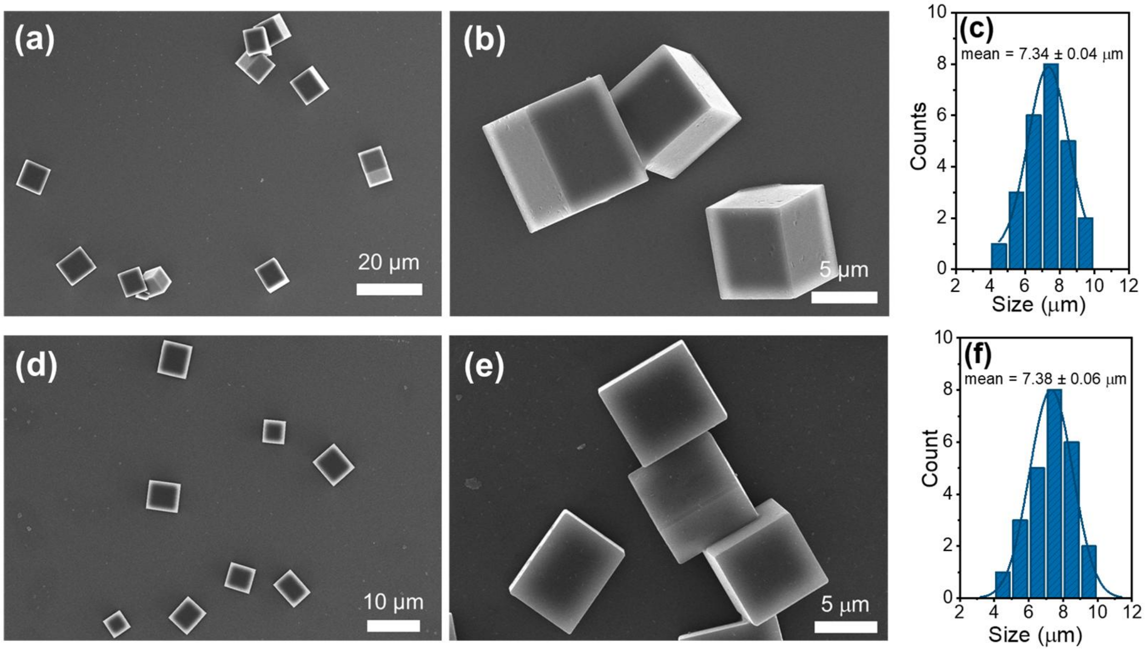

Figure S4. Scanning electron microscopy (SEM) images and corresponding histograms showing the length and width distributions of (a–c) pristine fullerene cubes (FC) and (d–f) oxidized fullerene cubes (FC_{ox}).

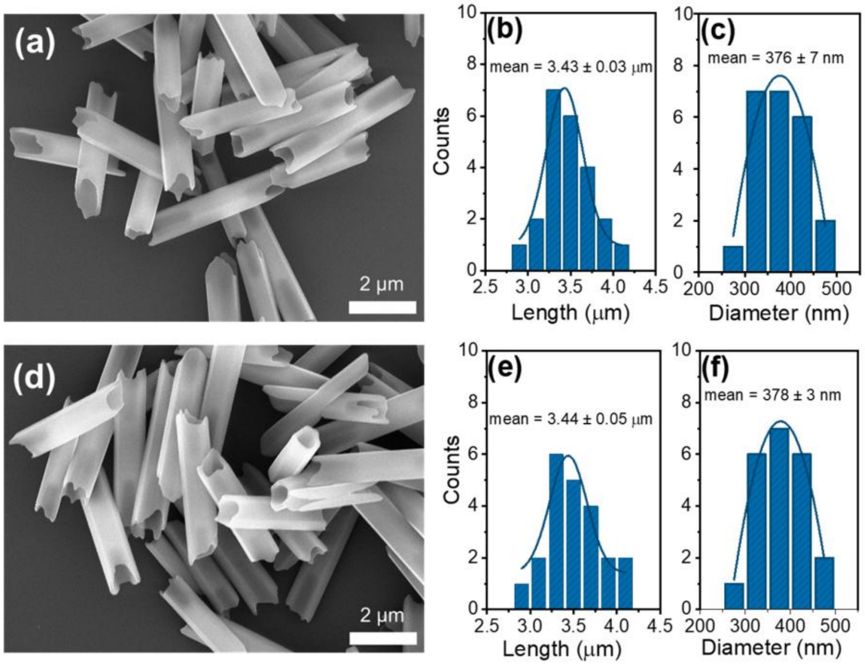

Figure S5. Scanning electron microscopy (SEM) images and corresponding histograms showing the length and diameter distributions of (a–c) pristine fullerene nanotubes (FNT) and (d–f) oxidized fullerene nanotubes (FNT_{ox}).

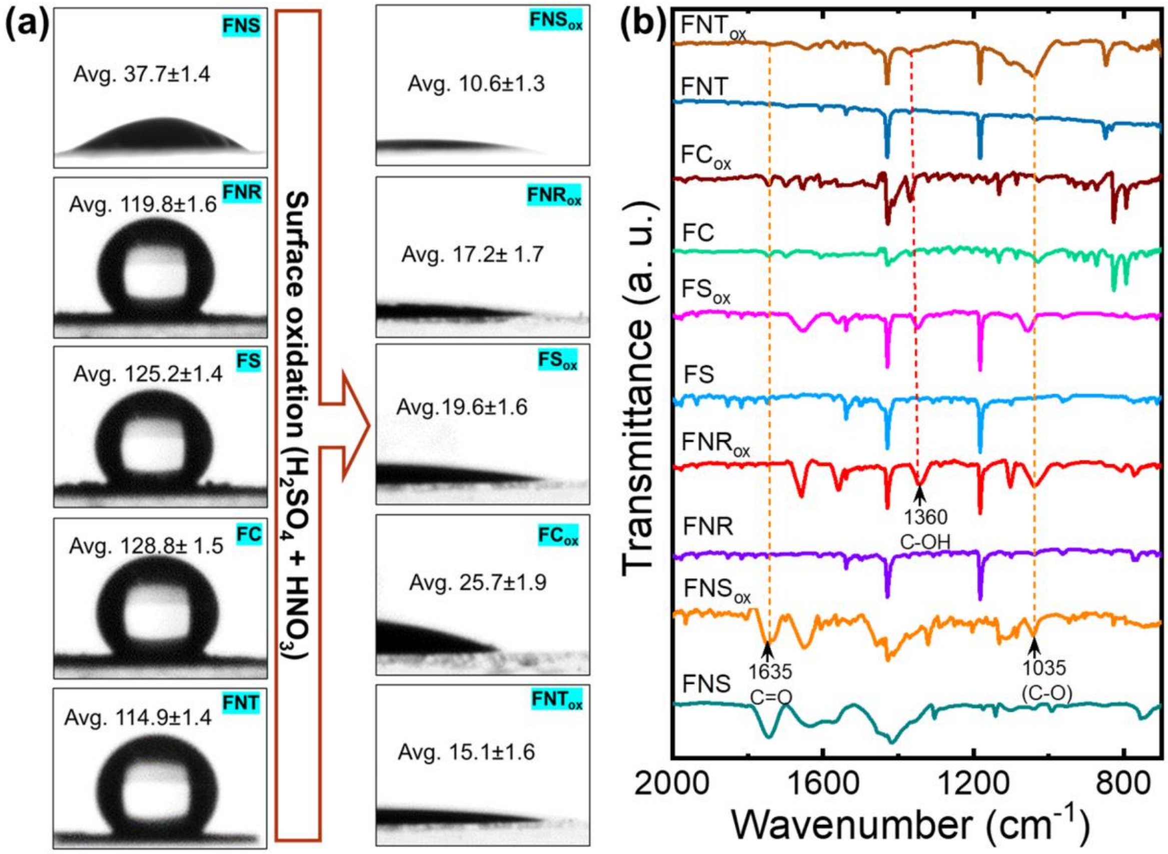

Figure S6. (a) Contact angle measurements and (b) FTIR spectra of pristine and oxidized fullerene assemblies, including FNS (nanospheres), FNR (nanorods), FS (nanosheets), FC (cubes), and FNT (nanotubes). The name of the materials was written on each image and spectrum.

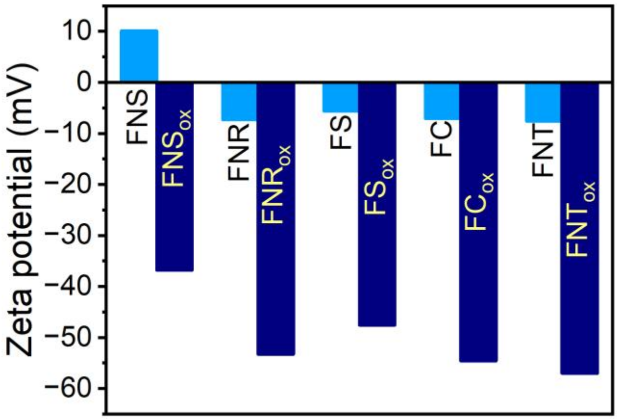

Figure S7. Zeta potential of pristine fullerene assemblies (FNS, FNR, FS, FC, FNT) and their oxidized forms (FNS_{ox}, FNR_{ox}, FS_{ox}, FC_{ox}, and FNT_{ox}).

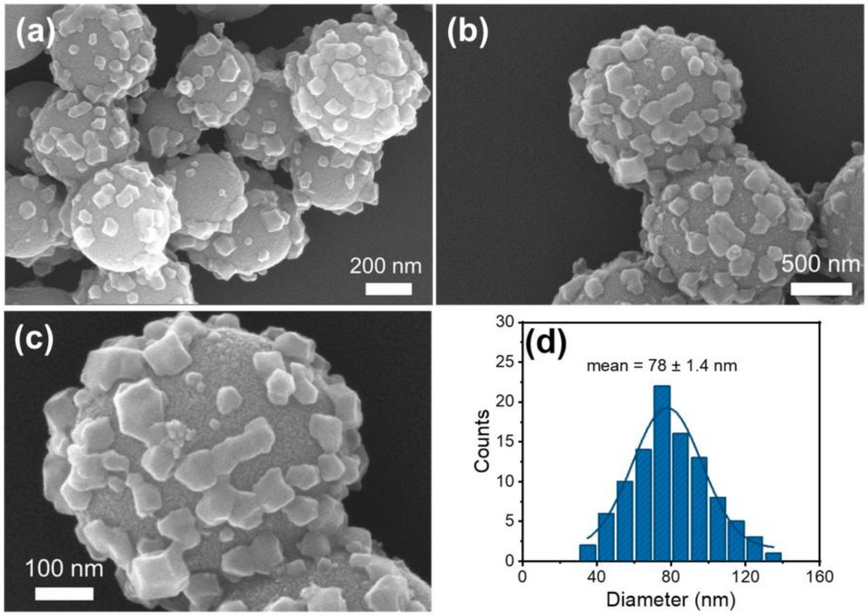

Figure S8. Scanning electron microscopy (SEM) images of (a-c) MOF on fullerene nanosphere (MOFOF-NS), along with the corresponding (d) size distribution histogram of the MOF particles.

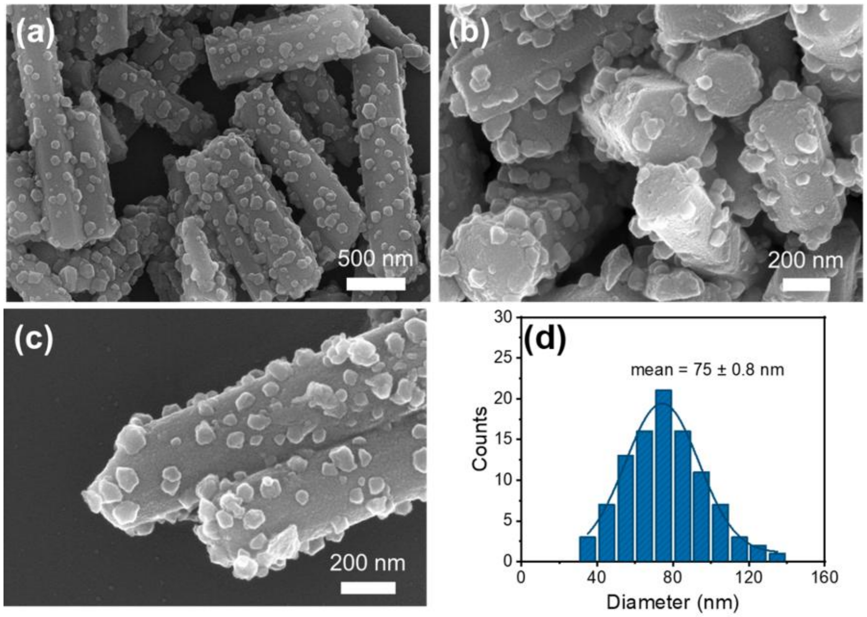

Figure S9. Scanning electron microscopy (SEM) images of (a-c) MOF on fullerene nanorod (MOFOF-NR), along with the corresponding (d) size distribution histogram of the MOF particles.

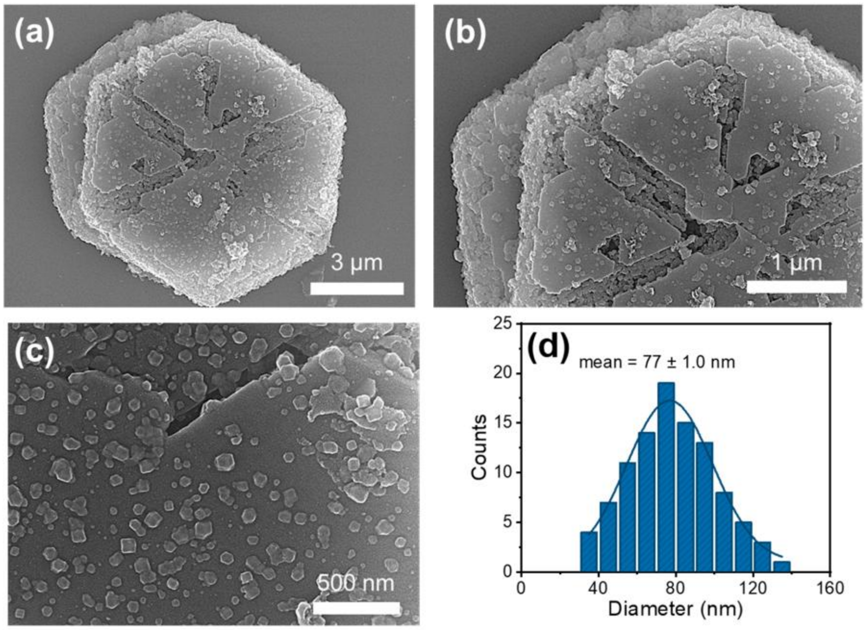

Figure S10. Scanning electron microscopy (SEM) images of (a-c) MOF on fullerene sheet (MOFOF-S), along with the corresponding (d) size distribution histogram of the MOF particles.

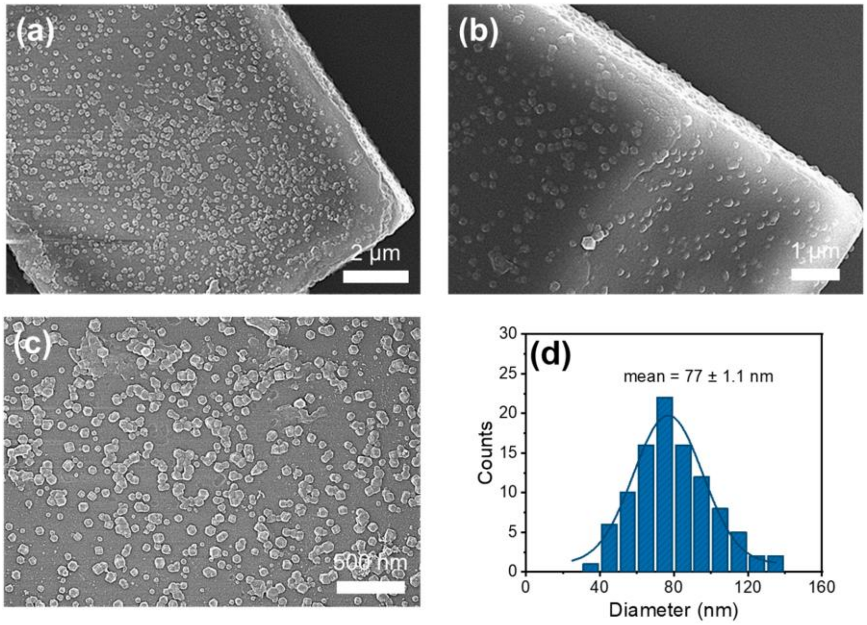

Figure S11. Scanning electron microscopy (SEM) images of (a-c) MOF on fullerene cube (MOFOF-C), along with the corresponding (d) size distribution histogram of the MOF particles.

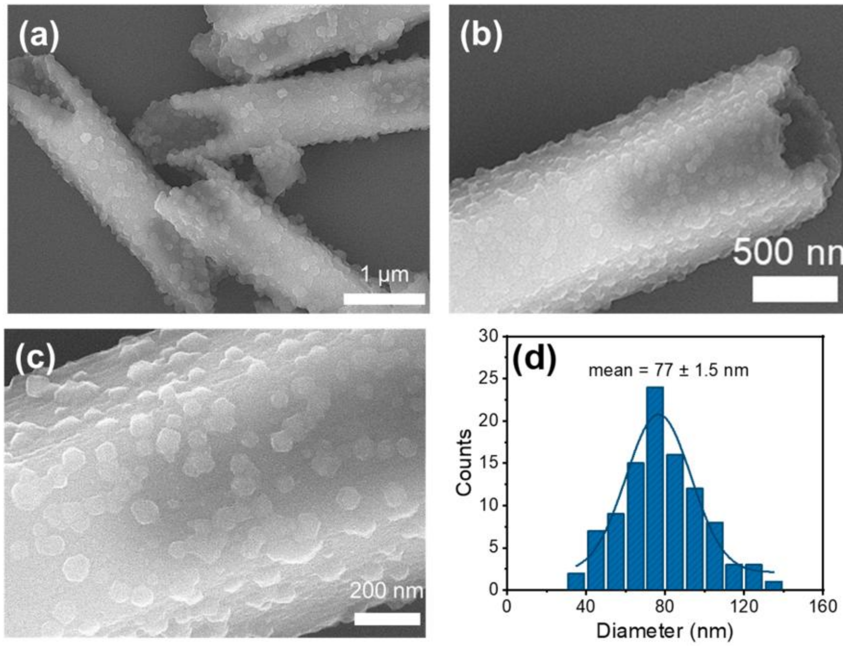

Figure S12. Scanning electron microscopy (SEM) images of (a-c) MOF on fullerene nanotube (MOFOF-NT), along with the corresponding (d) size distribution histogram of the MOF particles.

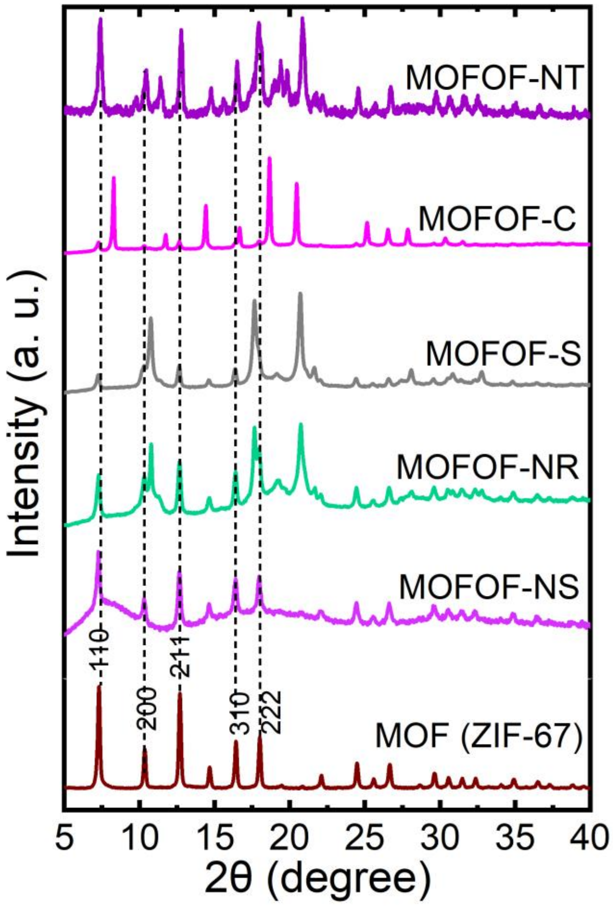

Figure S13. XRD patterns of MOF (ZIF-67), MOFOF-NS, MOFOF-NR, MOFOF-S, MOFOF-C, and MOFOF-NT.

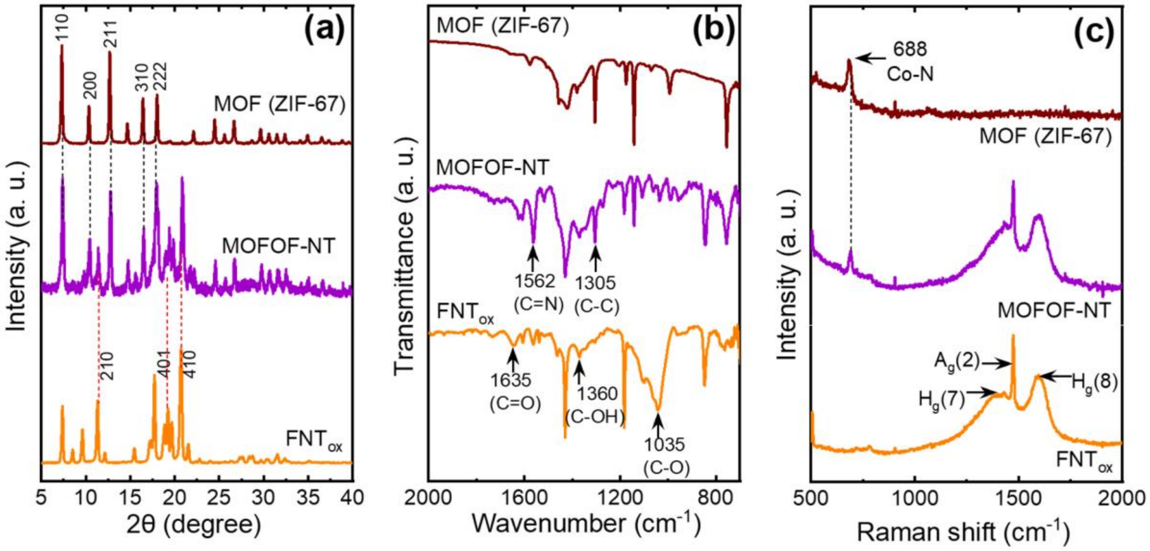

Figure S14. Relative (a) XRD patterns (b) FTIR and (c) Raman Spectrum of FNT$_{ox}$ MOFOF-NT and the MOF (ZIF-67).

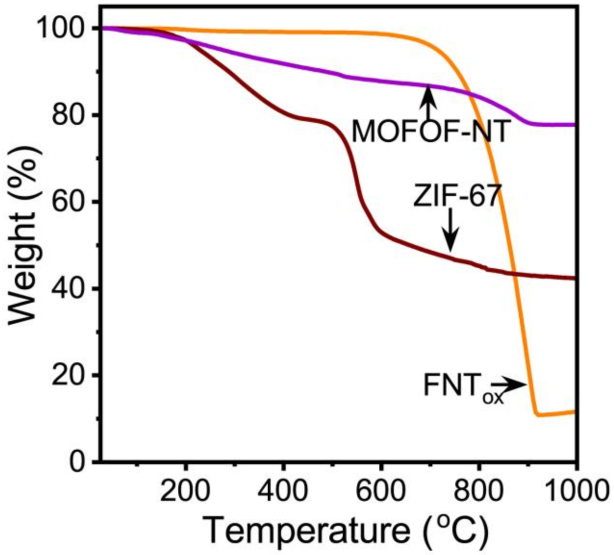

Figure S15. Thermogravimetric profile of FNT$_{ox}$, MOF (ZIF-67), and MOFOF-NT.

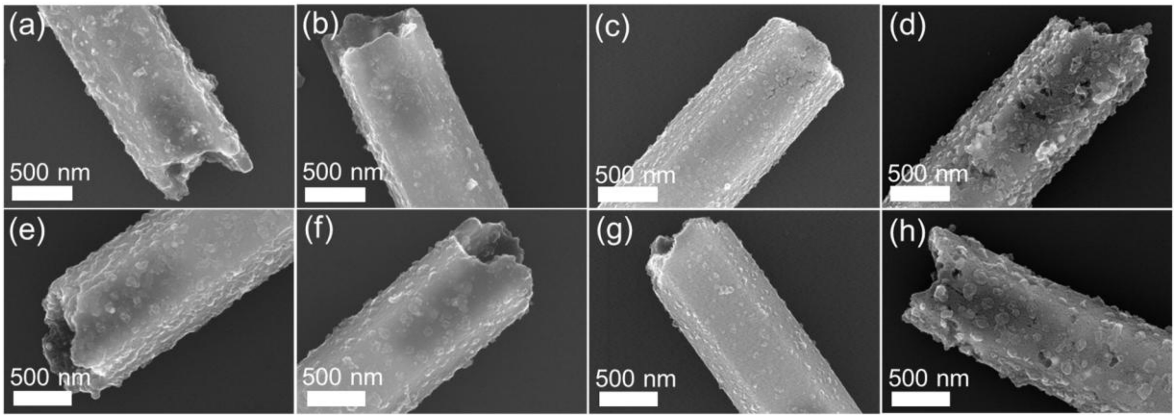

Figure S16. Scanning electron microscopy (SEM) images of MOFOF-derived materials carbonized at different temperatures: (a) Co@CT-800, (b) Co@CT-900, (c) Co@CT-1000, and (d) Co@CT-1100. Corresponding nitrogen-doped samples after ammonia treatment: (e) Co–N@CT-800, (f) Co–N@CT-900, (g) Co–N@CT-1000, and (h) Co–N@CT-1100.

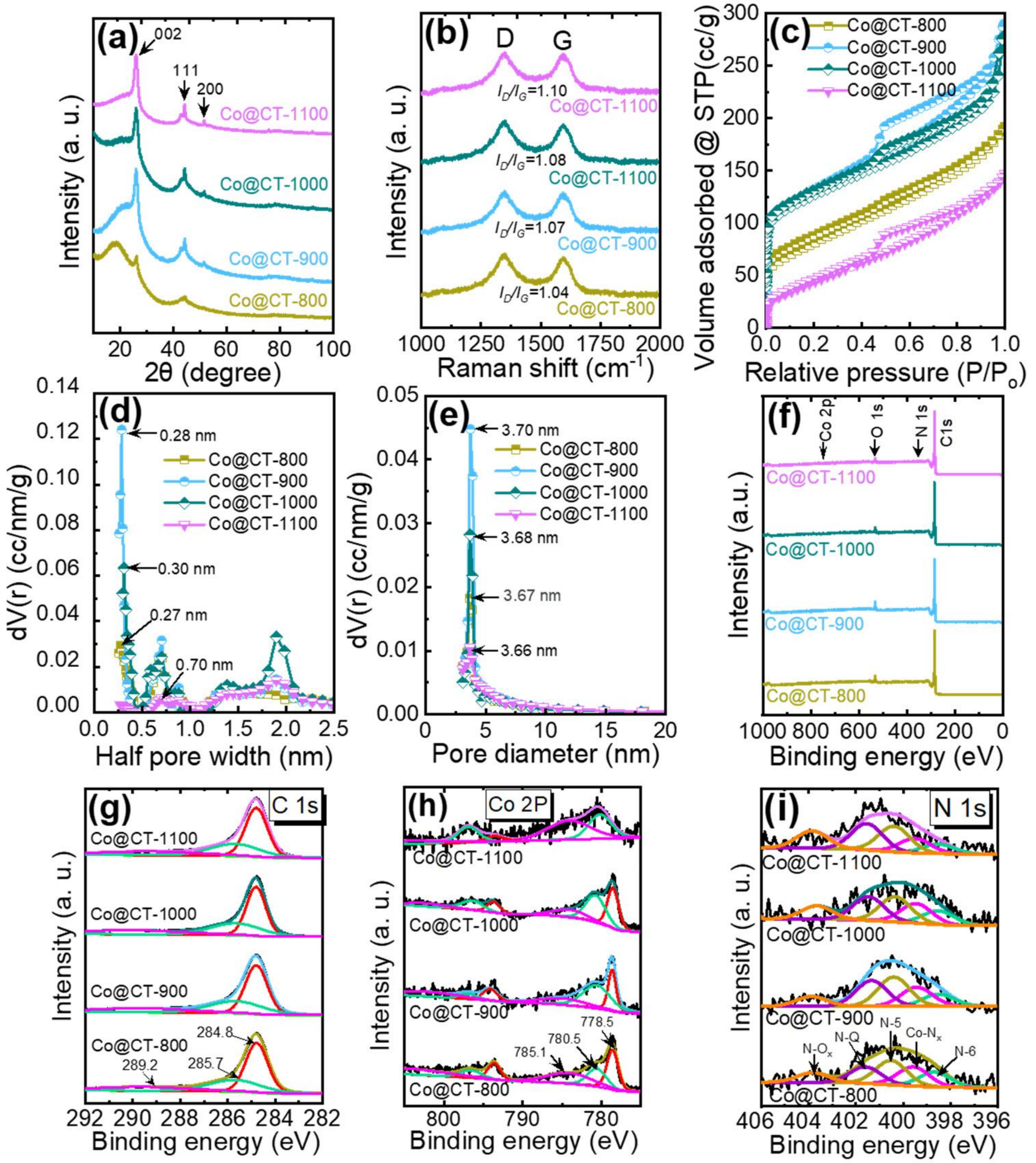

Figure S17. Physiochemical characterization of Co@CT–800, Co@CT–900, Co@CT–1000, and Co@CT–1100 materials: (a) Powder X-ray diffraction (XRD) patterns; (b) Raman scattering spectra; (c) Nitrogen adsorption–desorption isotherms; (d) Pore size distribution calculated by the DFT method; (e) Pore size distribution calculated by the BJH method; (f) XPS survey spectra and corresponding surface composition; (g–i) High-resolution XPS spectra of C 1s, Co 2p, and N 1s, respectively.

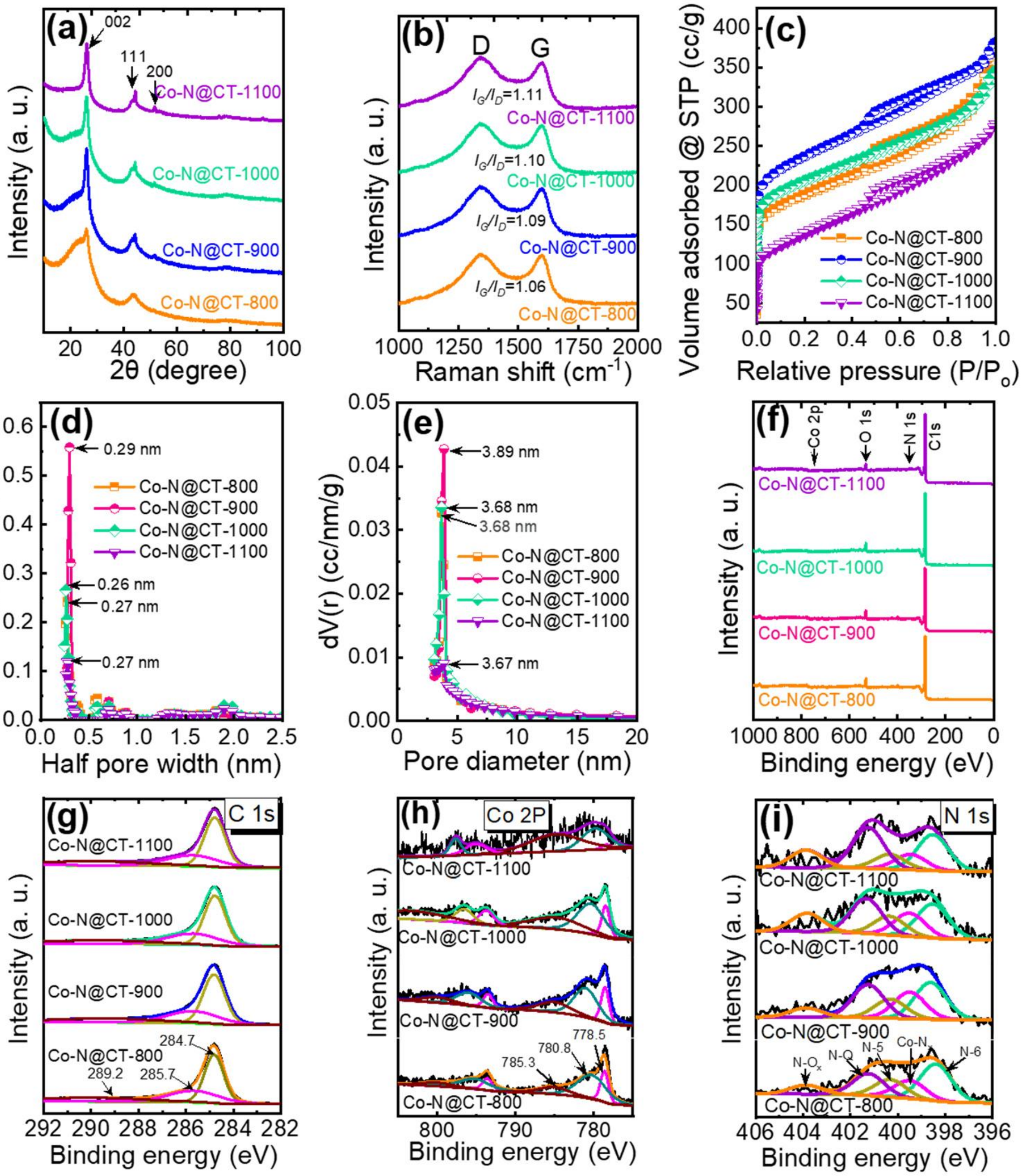

Figure S18. Physiochemical characterization of Co−N@CT−800, Co −N@CT−900, Co −N@CT−1000, and Co −N@CT−1100 materials: (a) Powder X-ray diffraction (XRD) patterns; (b) Raman scattering spectra; (c) Nitrogen adsorption–desorption isotherms; (d) Pore size distribution calculated by the DFT method; (e) Pore size distribution calculated by the BJH method; (f) XPS survey spectra and corresponding surface composition; (g–i) High-resolution XPS spectra of C 1s, Co 2p, and N 1s, respectively.

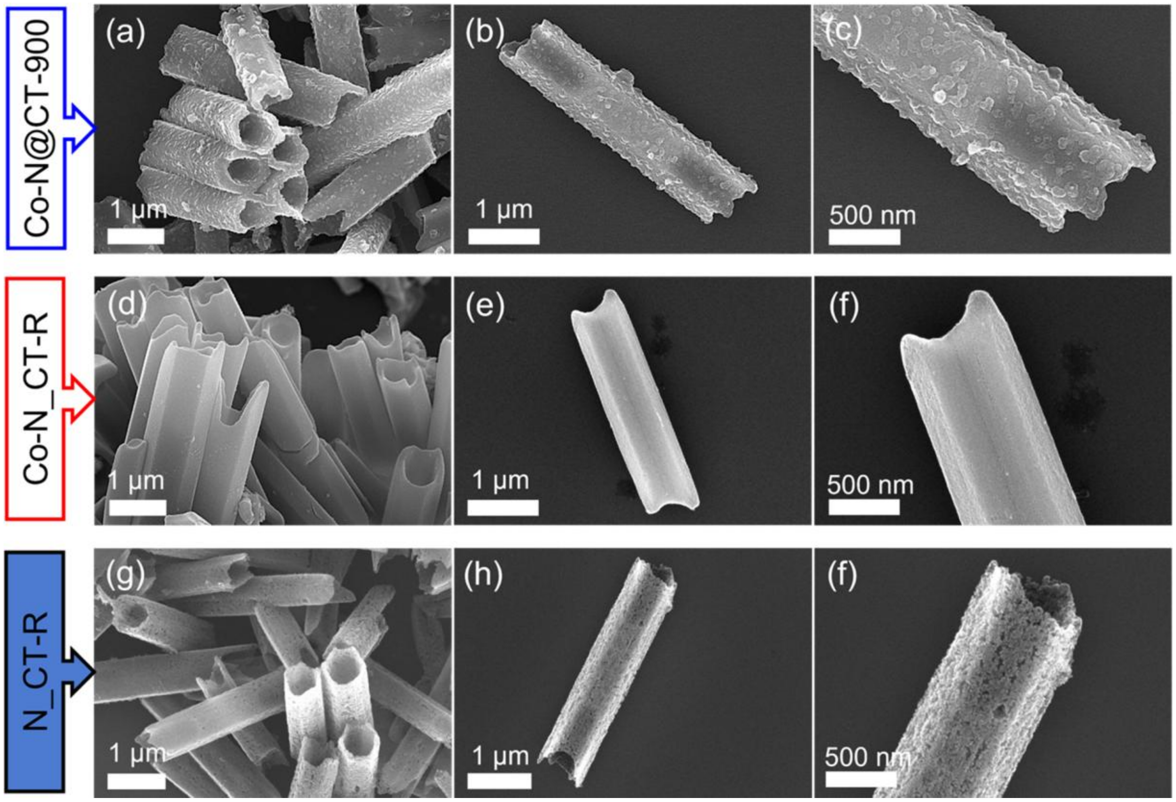

Figure S19. Scanning electron microscopy (SEM) images of the synthesized materials (a-c) Co-N@CT-900; (d-f) Co-N_CT-R; and (g-i) N_CT-R.

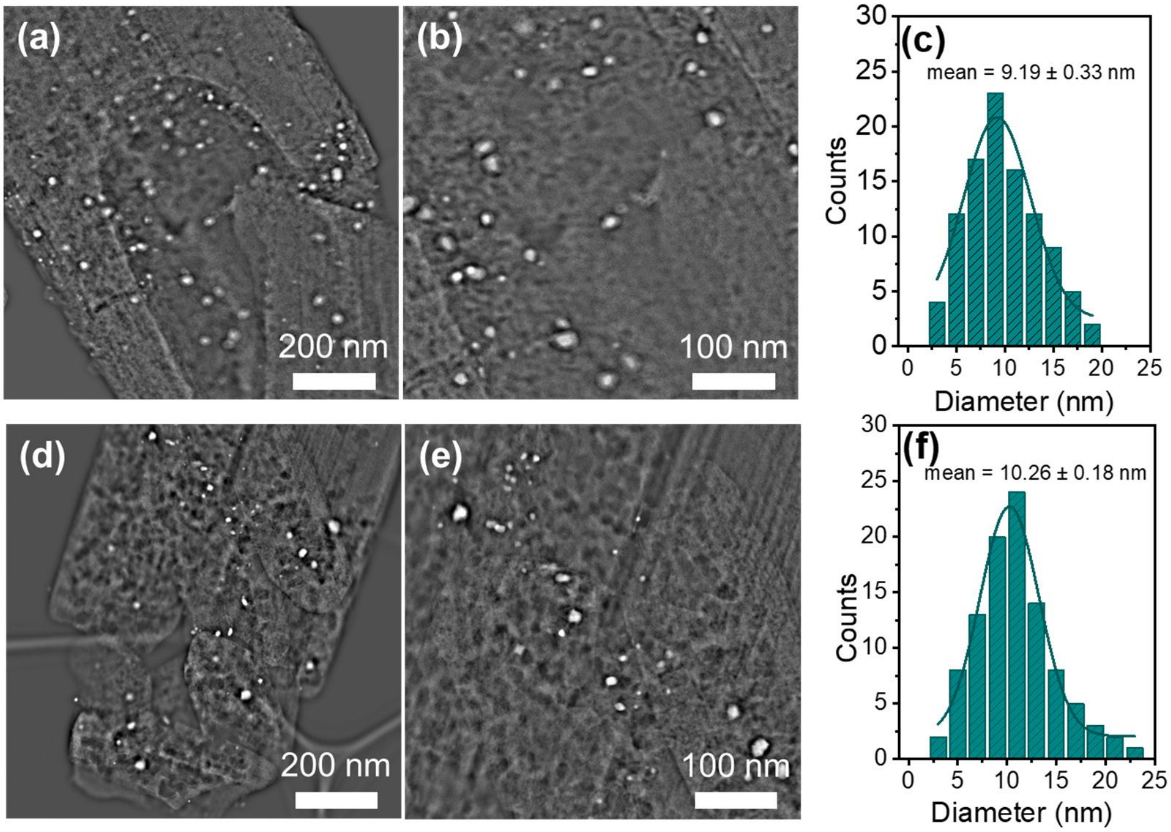

Figure S20. Scanning transmission electron microscopy (STEM) images and corresponding size distribution profiles of cobalt nanoparticles derived from MOFOF: (a–c) Co–N@CT–900, and the cobalt-impregnated reference material: (d–f) Co–N_CT–R.

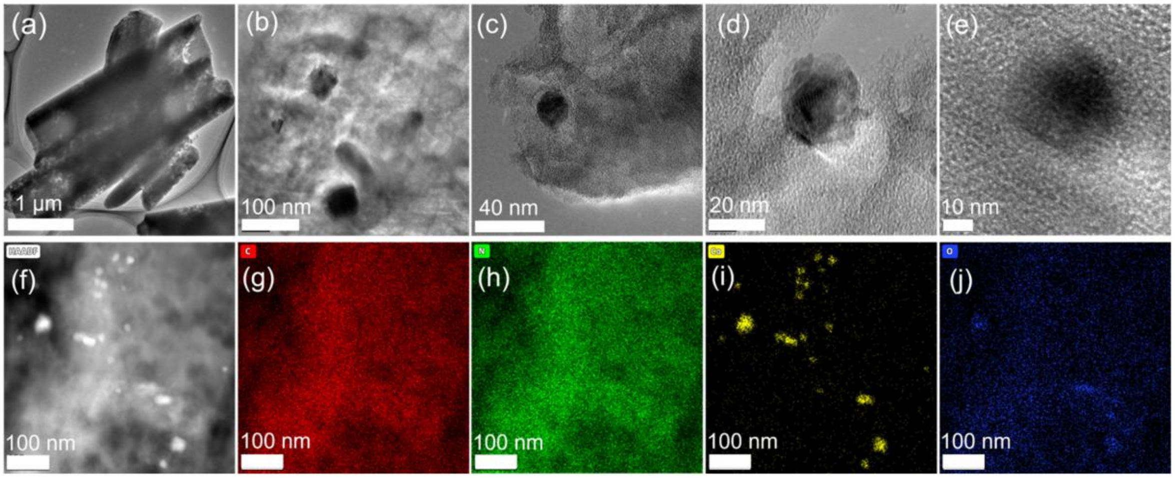

Figure S21. Electron microscopy analysis of Co-N_CT-R. (a-e) Transmission electron microscopy (TEM) images; (f) Scanning transmission electron microscopy (STEM) image; EDX elemental mapping for Carbon (g), Nitrogen (h), (i) Cobalt, and (j) Oxygen.

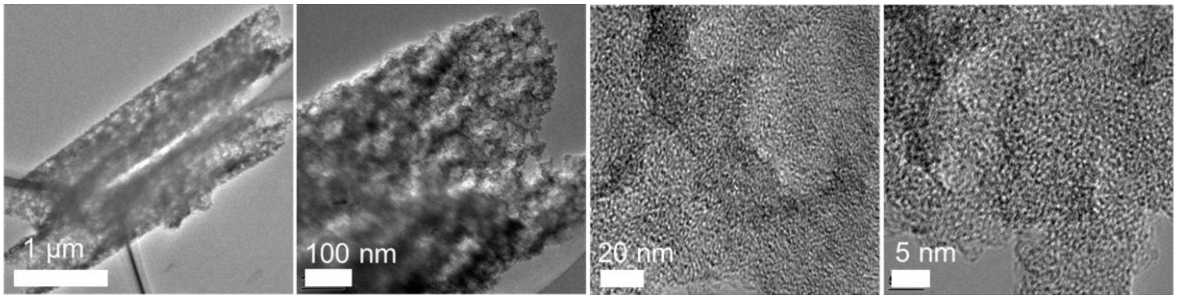

Figure S22. Transmission electron microscopy (TEM) images of N_CT-R at different magnifications.

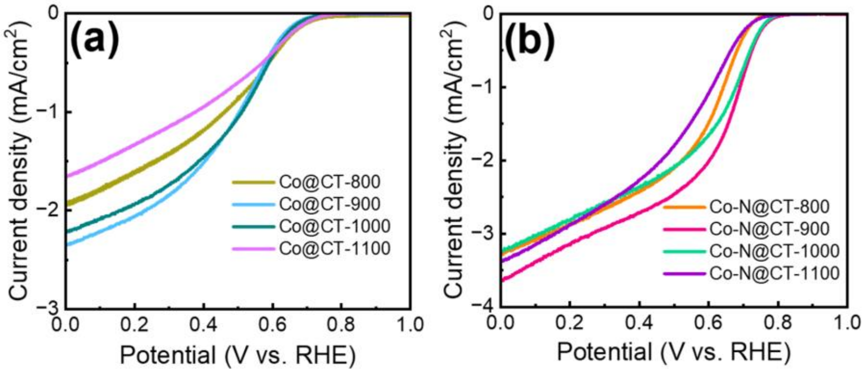

Figure S23. Linear sweep voltammetry (LSV) curves of (a) before nitrogen-doping and (b) after nitrogen-doping in O_2-saturated conditions in 0.1 M H_2SO_4 electrolyte recorded at a rotation rate of 1600 rpm in a rotating ring disk electrode (RRDE).

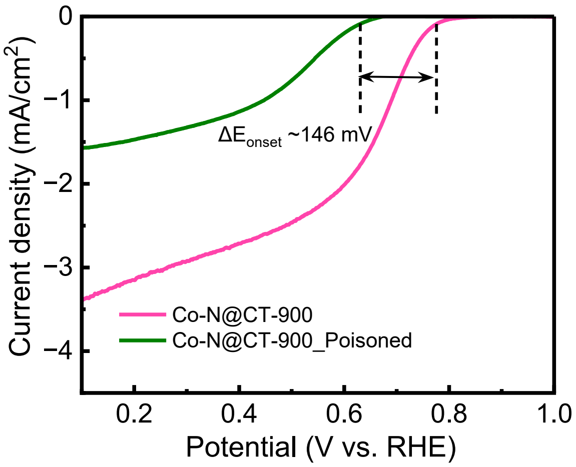

Figure S24. Electrochemical oxygen reduction reaction (ORR) activity of Co–N@CT–900 catalyst in 0.1 M H_2SO_4 and 0.1 M H_2SO_4+10 mM KSCN electrolyte solution recorded at 1600 rpm.

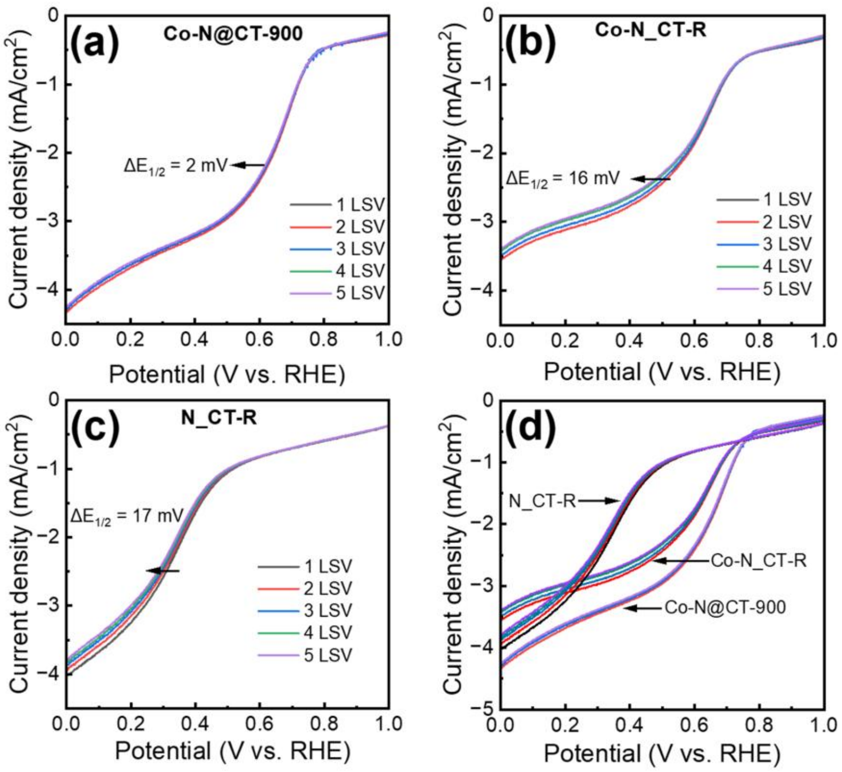

Figure S25. Durability performance of the (a) Co-N@CT-900, (b) Co-N_CT-R, (c) N_CT-R and (d) comparison of the catalysts were conducted by performing CV from 1 V to 0 V vs. RHE in O_2-saturated in 0.1 M H_2SO_4 electrolyte at room temperature. The protocol involved 20 CV cycles followed by one LSV measurement, repeating this sequence. The black line represents the initial LSV and the purple line represents the LSV after 100 CV cycles, the change in $E_{1/2}$ after 100 CV cycles.

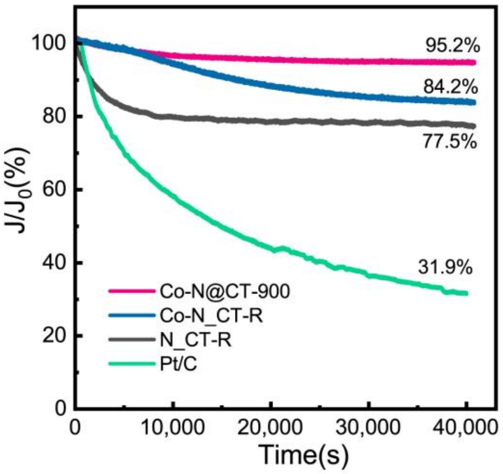

Figure S26. Relative chronoamperometric response of the studied materials and the conventional ORR catalysts Pt/C.

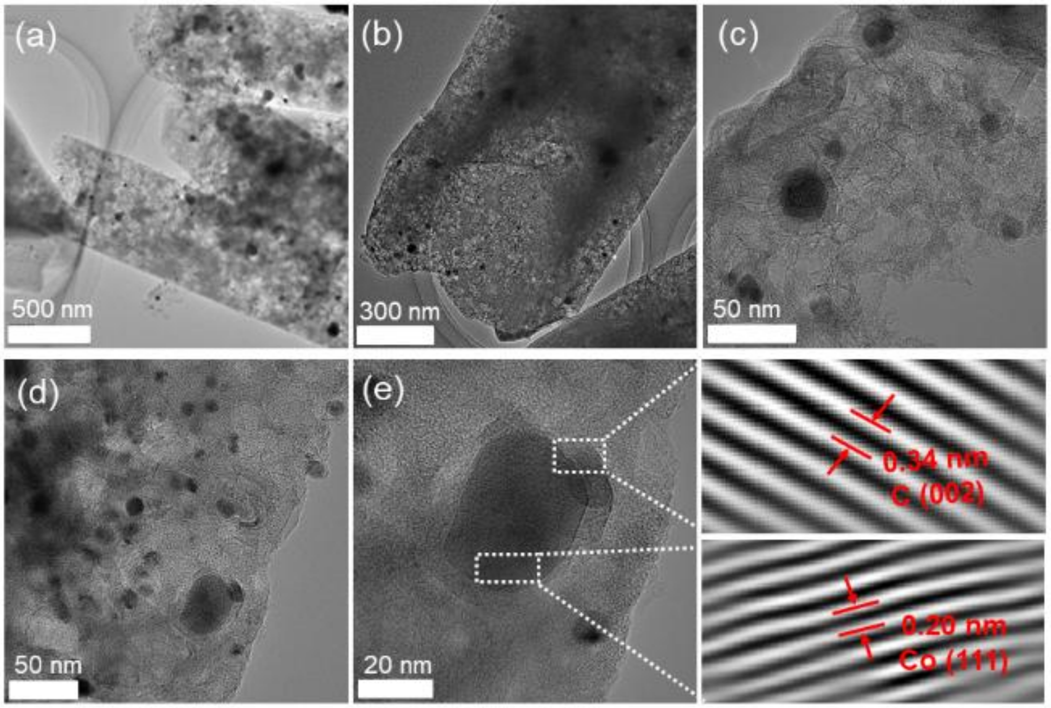

Figure S27. Transmission electron microscopy (TEM) characterization of Co–N@CT–900 after catalysis. (a–b) TEM images at increasing magnifications reveal a well-developed porous carbon matrix; (c-e) high-resolution TEM (HRTEM) images display well-defined lattice fringes corresponding to graphitic carbon and metallic cobalt, indicating crystalline domains and structural integrity of the catalyst after catalysis.

REFERENCES

1. Ariga, K. Liquid–Liquid Interfacial Nanoarchitectonics. *Small* **2024**, *20*, 2305636. DOI: 10.1002/smll.202305636
2. Ariga, K.; Shrestha, L. K. Zero-to-One (or More) Nanoarchitectonics: How to Produce Functional Materials from Zero-Dimensional Single-Element Unit, Fullerene. *Mater Adv*. **2021**, *2*, 582-597. DOI: 10.1039/d0ma00744g
3. Baskar, A. V.; Benzigar, M. R.; Talapaneni, S. N.; Singh, G.; Karakoti, A. S.; Yi, J.; Al-Muhtaseb, A. A.; Ariga, K.; Ajayan, P. M.; Vinu, A. Self‐Assembled Fullerene Nanostructures: Synthesis and Applications. *Adv. Funct. Mater.* **2022**, *32*, 2106924. DOI: 10.1002/adfm.202106924
4. Kumar, G. S.; Shrestha, R. G.; Ji, Q.; Hill, J. P.; Ariga, K.; Acharya, S.; Shrestha, L. K. Hierarchical Heterostructure of Ag-Nanoparticle Decorated Fullerene Nanorods (Ag–FNRs) as an Effective Single Particle Freestanding SERS Substrate. *Phys. Chem. Chem. Phys.* **2018**, *20*(27),18873-18878. DOI: 10.1039/C8CP02779J
5. Shrestha, L. K.; Yamauchi, Y.; Hill, J. P.; Miyazawa, K. I.; Ariga, K. Fullerene Crystals with Bimodal Pore Architectures Consisting of Macropores and Mesopores. *J. Am. Chem. Soc.* **2013**, *135*(2), 586-589. DOI: 10.1021/ja3108752
6. Bairi, P.; Minami, K.; Nakanishi, W.; Hill, J. P.; Ariga, K.; Shrestha, L. K. Hierarchically Structured Fullerene C_{70} Cube for Sensing Volatile Aromatic Solvent Vapors. *ACS nano* **2016**, *10*(7), 6631-6637. DOI: 10.1021/acsnano.6b01544
7. Shrestha, L. K.; Shrestha, R. G.; Yamauchi, Y.; Hill, J. P.; Nishimura, T.; Miyazawa, K. I.; Kawai, T.; Okada, S.; Wakabayashi, K.; Ariga, K. Nanoporous Carbon Tubes from Fullerene Crystals as the Π‐Electron Carbon Source. *Angew. Chem.* **2015**,*127*(3), 965-969. DOI: 10.1002/ange.201408856
8. Chen, G.; Sciortino, F.; Takeyasu, K.; Nakamura, J.; Hill, J. P.; Shrestha, L. K.; Ariga, K. Hollow Spherical Fullerene Obtained by Kinetically Controlled Liquid-Liquid Interfacial Precipitation. *Chem. Asian J.* **2022**, *17*, e202200756. DOI: 10.1002/asia.202200756

9. Shrestha, L. K.; Wei, Z.; Subramaniam, G.; Shrestha, R. G.; Singh, R.; Sathish, M.; Ma, R.; Hill, J. P.; Nakamura, J.; Ariga, K. Nanoporous Hollow Carbon Spheres Derived from Fullerene Assembly as Electrode Materials for High-Performance Supercapacitors. *Nanomaterials* **2023**, *13* (5), 946. DOI: 10.3390/nano13050946
10. Zou, H.; Shu, S.; Yang, W.; Chu, Y. C.; Cheng, M.; Dong, H.; Liu, H.; Li, F.; Hu, J.; Wang, Z.; Liu, W. Steering Acidic Oxygen Reduction Selectivity of Single-Atom Catalysts Through the Second Sphere Effect. *Nat. Commun*. **2024**, *15*(1), 10818. DOI: 10.1038/s41467-024-55116-x
11. Chen, S.; Xu, Q.; Sun, H.; Ge, L.; Huang, D.; Zhang, Z.; Qiao, Y.; Tong, X.; Fan, W. Cobalt-Embedded Nitrogen-Doped Carbon Nanosheets for Oxygen Reduction Reaction under Alkaline and Acidic Media. *ACS App. Nano Mater.* **2024**, *7*(13), 15710-15719. DOI: 10.1021/acsanm.4c02768
12. Irmawati, Y.; Balqis, F.; Destyorini, F.; Adios, C. G.; Yudianti, R.; Iskandar, F.; Sumboja, A. Cobalt Nanoparticles Encapsulated with N-Doped Bamboo-Like Carbon Nanofibers as Bifunctional Catalysts for Oxygen Reduction/Evolution Reactions in a Wide pH Range. *ACS App. Nano Mater.* **2023**, *6*(4), 2708–2718. DOI: 10.1021/acsanm.2c05091
13. Gong, M.; Mehmood, A.; Ali, B.; Nam, K. W.; Kucernak, A. Oxygen Reduction Reaction Activity in Non-Precious Single-Atom (M–N/C) Catalysts— Contribution of Metal and Carbon/Nitrogen Framework-Based Sites. *ACS Catal.* **2023**, *13*(10), 6661-6674. DOI: 10.1021/acscatal.3c00356
14. Chen, S.; Luo, T.; Li, X.; Chen, K.; Fu, J.; Liu, K.; Cai, C.; Wang, Q.; Li, H.; Chen, Y.; Ma, C. Identification of the Highly Active Co–N4 Coordination Motif for Selective Oxygen Reduction to Hydrogen Peroxide. *J. Am. Chem. Soc.***2022**, *144*(32), 14505-14516. DOI: 10.1021/jacs.2c01194
15. Wang, X. X; Cullen, D. A.; Pan, Y. T.; Hwang, S.; Wang, M.; Feng, Z.; Wang, J.; Engelhard, M. H.; Zhang, H.; He, Y.; Shao, Y. Nitrogen-Coordinated Single Cobalt Atom Catalysts for Oxygen Reduction in Proton Exchange Membrane Fuel Cells. *Adv. Mater*. **2018,** *30*(11), 1706758. DOI: 10.1002/adma.201706758

16. Han, Y.; Wang, Y. G.; Chen, W.; Xu, R.; Zheng, L.; Zhang, J.; Luo, J.; Shen, R. A.; Zhu, Y.; Cheong, W. C.; Chen, C. Hollow N-Doped Carbon Spheres with Isolated Cobalt Single Atomic Sites: Superior Electrocatalysts for Oxygen Reduction. *J. Am. Chem. Soc.* 2017, *139*(48), 17269-17272. DOI:10.1021/jacs.7b10194
17. Subramanian, P.; Mohan, R.; Schechter, A. Unraveling the Oxygen-Reduction Sites in Graphitic-Carbon Co–N–C-Type Electrocatalysts Prepared by Single-Precursor Pyrolysis. *ChemCatChem*. **2017**, *9*(11):1969-1978. DOI: 10.1002/cctc.201700324
18. Yan, X.; Liu, K.; Wang, T.; You, Y.; Liu, J.; Wang, P.; Pan, X.; Wang, G.; Luo, J.; Zhu, J. Atomic Interpretation of High Activity on Transition Metal and Nitrogen-Doped Carbon Nanofibers for Catalyzing Oxygen Reduction. *J. Mater. Chem. A*. **2017**, *5*(7):3336-3345. DOI: 10.1039/c6ta09462g
19. Zhang, Z.; Dou, M.; Liu, H.; Dai, L.; Wang, F. A Facile Route to Bimetal and Nitrogen-Codoped 3D Porous Graphitic Carbon Networks for Efficient Oxygen Reduction. *Small* **2016**, *12*(31), 4193-4199. DOI:10.1002/smll.201601617
20. Fu, X.; Choi, J. Y.; Zamani, P.; Jiang, G.; Hoque, M. A.; Hassan, F. M.; Chen, Z. Co–N Decorated Hierarchically Porous Graphene Aerogel for Efficient Oxygen Reduction Reaction in Acid. *ACS App. Mater. Inter*. **2016**, *8*(10), 6488-6495. DOI: 10.1021/acsami.5b12746
21. You, B.; Jiang, N.; Sheng, M.; Drisdell, W. S.; Yano, J.; Sun, Y. Bimetal–Organic Framework Self-Adjusted Synthesis of Support-Free Nonprecious Electrocatalysts for Efficient Oxygen Reduction. *ACS Catal*. **2015**, *5*(12):7068-7076. DOI: 10.1021/acscatal.5b02325
22. Liang, H. W.; Wei, W.; Wu, Z. S.; Feng, X.; Mullen, K. Mesoporous Metal–Nitrogen-Doped Carbon Electrocatalysts for Highly Efficient Oxygen Reduction Reaction. *J. Am. Chem. Soc.***2013**, *135*(43), 16002-16015. DOI: 10.1021/ja407552k
23. Liu, H.; Jiang, L.; Khan, J.; Wang, X.; Xiao, J.; Zhang, H.; Xie, H.; Li, L.; Wang, S.; Han, L. Decorating Single-Atomic Mn Sites with FeMn Clusters to Boost Oxygen Reduction Reaction. *Angew. Chem.***2023**, *135*(3), 202214988. DOI: 10.1002/anie.202214988

24. Yang, G.; Zhu, J.; Yuan, P.; Hu, Y.; Qu, G.; Lu, B. A.; Xue, X.; Yin, H.; Cheng, W.; Cheng, J.; Xu, W. Regulating Fe-Spin State by Atomically Dispersed Mn-N in Fe-NC Catalysts with High Oxygen Reduction Activity. *Nat. Commun.***2021**, *12*(1), 1734. DOI: 10.1038/s41467-021-21919-5
